# Exploring Structural Diversity of Microbe Secondary Metabolites Using OSMAC Strategy: A Literature Review

**DOI:** 10.3389/fmicb.2019.00294

**Published:** 2019-02-26

**Authors:** Rui Pan, Xuelian Bai, Jianwei Chen, Huawei Zhang, Hong Wang

**Affiliations:** ^1^School of Pharmaceutical Sciences, Zhejiang University of Technology, Hangzhou, China; ^2^College of Life and Environmental Sciences, Hangzhou Normal University, Hangzhou, China

**Keywords:** OSMAC strategy, microbe secondary metabolite, structural diversity, medium composition, co-cultivation, epigenetic modification

## Abstract

Microbial secondary metabolites (MSMs) have played and continue to play a highly significant role in the drug discovery and development process. Genetically, MSM chemical structures are biologically synthesized by microbial gene clusters. Recently, however, the speed of new bioactive MSM discovery has been slowing down due to consistent employment of conventional cultivation and isolation procedure. In order to alleviate this challenge, a number of new approaches have been developed. The strategy of one strain many compounds (OSMAC) has been shown as a simple and powerful tool that can activate many silent biogenetic gene clusters in microorganisms to make more natural products. This review highlights important and successful examples using OSMAC approaches, which covers changing medium composition and cultivation status, co-cultivation with other strain(s), adding enzyme inhibitor(s) and MSM biosynthetic precursor(s). Available evidences had shown that variation of cultivation condition is the most effective way to produce more MSMs and facilitate the discovery of new therapeutic agents.

## Introduction

Microbial secondary metabolites (MSMs) have been recognized as the primary source of new compounds for drug discovery and development (Gunatilaka, 2006; Rateb et al., 2011b; Deng et al., 2013). Traditional chemical investigation of microorganism mainly focuses on extraction and isolation of structurally and highly active compounds from fermentation broth and mycelium. However, these processes are becoming inefficient due to high rate of the re-discovery of known MSMs. It is commonly believed that a large portion of microbial gene clusters are silenced under standard fermentation conditions (Scherlach and Hertweck, 2009; Wasil et al., 2013). By mining microbial genome and targeting biosynthetic gene clusters of MSM, researchers can exploit the potential of microbes in a more objective way, such as knocking down, introduction or heterologous expression of microbial genes, regulation of promoters, induction of mutations, or changing cultivation conditions to stimulate MSM genes expression (Schneider et al., 2008). Variation of cultivation condition has been deemed to be the simplest and most effective strategy, which is termed as “one strain many compounds (OSMAC)" by professor Zeeck and co-workers (Bode et al., 2002). On basis of extensive literature search, important and successful examples using OSMAC strategy are summarized in this review, which consists of variation of medium, changing cultivation condition, co-cultivation with other strain(s), adding epigenetic modifier(s) or biosynthetic precursor(s).

## Variation of Medium

Culture medium has a greater effect not only on microbe growth but also on metabolism. It has been reported that C/N ratio, salinity, and metal ion can regulate the degree and pattern of MSM gene expression and result in production of various secondary metabolites.

### Medium Composition

Generally, carbon and nitrogen sources are major components in the culture medium. The carbon source not only provides the basis for building biomass and represents the source of energy for all heterotrophs but also delivers carbon units for secondary metabolites. The nitrogen source is required for the synthesis of essential proteins and nucleic acids, and likewise *N*-containing units for secondary metabolites. The type of used carbon and nitrogen sources is known to have a significant influence on microbial secondary metabolism (Ruiz et al., 2009; Singh et al., 2017). Furthermore, the C/N ratio is one of important factors that affect fermentation products (Karakoç and Aksöz, 2004; Brzonkalik et al., 2012; Dinarvand et al., 2013). Notably, the consumption of carbon and nitrogen-based medium components can greatly affects the pH of the cultivation broth, e.g., by formation of organic acids or the accumulation of basic ammonium. Thus, microorganisms cultured in medium containing different components may exhibit differently adapted metabolism and express specific sets of biosynthetic genes, which produced a differential biosynthesis of specialized metabolites (Ma et al., 2009).

One marine-derived strain *Asteromyces cruciatus* 763 was shown to produce a new pentapeptide lajollamide A (**1**), when cultivated in the Czapek-Dox broth contained arginine solely as nitrogen source rather than NaNO_3_, which was missed in the normal Czapek-Dox medium (Gulder et al., 2012). One sediment-derived *Aspergillus niger* BRF-074 produced a novel furan ester derivative (**2**), a compound has toxicity acidity against HCT-116 cancer cell line (Uchoa et al., 2017), when cultivated in MPDB (malt peptone dextrose broth) medium. But this compound failed to appear in PDB (potato dextrose broth) or PDYB (potato dextrose yeast broth) media. A fungus *Aspergillus* sp. from Waikiki Beach (Honolulu, HI), generated six isotopically labeled metabolites (**3**–**8**) when grown on the deuterium-enriched Czapek broth (Wang et al., 2015a), whereas this strain was found to metabolite a novel prenylated indole alkaloid, waikialoid A (**9**) when cultivated in PDB medium. Bioassay results indicated that compound **9** possessed potent inhibitory effect on biofilm formation of *Candida albicans* with an IC_50_ value of 1.4 μM (Wang Q.X. et al., 2012).


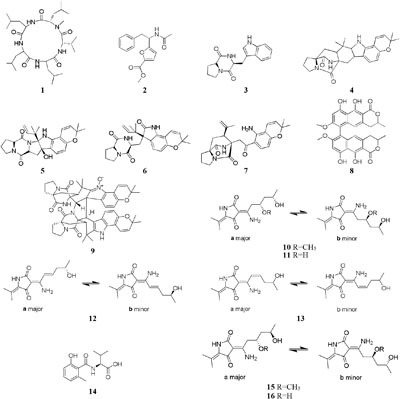



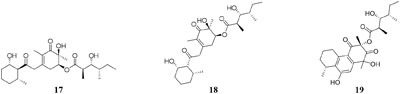


Five new polyketides (**10**–**14**) were detected in the crude extract of rice-based medium of a marine-derived *Cladosporium sphaerospermum* 2005-01-E3 (Wu et al., 2014). Another two new hybrid polyketides (**15**–**16**) were accessed when the same strain was fermented on the soybean flour (Yu et al., 2015). The organic extract of *Dothideomycete* sp. CRI7 was elaborated by four comparative medium. The strain growing in PDB made with potato tubers led to the isolation of azaphilone derivatives (**17**–**18**) and a novel tricyclic polyketide (**19**). Only compound **19** exhibited a broad spectrum of cytotoxic activities (Senadeera et al., 2012). It is interesting that MSM production by strain CR17 was sensitive to sources of potato and malt extract used for the preparation of PDB and Czapek malt media, respectively. Three new polyketides (**20**–**22**) were produced when strain CR17 was grown in PDB broth prepared from a commercial potato powder instead of fresh tubers of potato, while this strain produced several other compounds (**20**–**21** and **23**–**25)** in Czapek malt medium. Compound **24** exhibited cytotoxic activity against cancer cell lines MOLT-3, HuCCA-1and A549 with IC_50_ values of 17.4, 48.1, 46.5 μg/mL, respectively (Hewage et al., 2014). One fungus strain of *Fusarium tricinctum* isolated in Beni-Mellal, which can colonize the rhizomes of *Aristolochia paucinervis*, could afforded three new fusarielins (**26**–**28**). But these metabolites were not detected when cultivated in normal rice medium supplemented with fruit and vegetable juice. Bioassay results suggested that compound **26** possessed cytotoxic effect on human ovarian cancer cell line A2780 with an IC_50_ value of 12.5 μM (Hemphill et al., 2017). A new diketopiperazine (**29**) was isolated from *Eurotium rubrum* MPUC136 cultured by wheat medium, which displayed more powerful bioactivity than the Czapek-Dox agar medium, and shown to have cytotoxicity against B_16_ melanoma cell line with an IC_50_ value of 60 μM (Kamauchi et al., 2016).


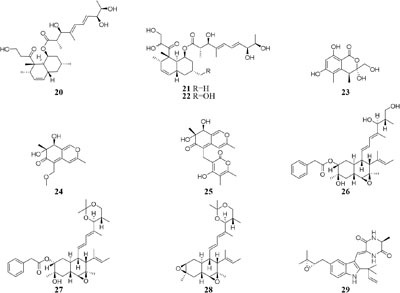


HPLC analysis of crude extracts of an actinomycete strain *Lentzea violacea* AS08 indicated different composition in three media including CYPS (casein yeast peptone), SCP-1 (starch casein peptone), and SC (starch casein) (Hussain et al., 2017). Only one new eudesmane sesquiterpenoid (**30**) and a new analog of virginiae butanolide E (**31**) were detected in SC medium, and compound **30** exhibited moderate cytotoxic effect on HCT-116 and A549 tumor cell lines with IC_50_ values of 19.2 and 22.3 μM, respectively. One rhizosphere fungus *Paraphaeosphaeria quadriseptata* produced a known C18 polyketide monocillin I together with several analogs when incubated in PDA medium constituted with tap water (Wijeratne et al., 2004). However, the same fungal strain could make six new trihydroxybenzene lactones, cytosporones F-I (**32**–**37**), when the tap water was changed as distilled water (Paranagama et al., 2007). Similarly, one new naphthalopyran compound (**38**), which possesses an unusual oxygenated aromatic structure with a lactone bridge, could be metabolized by the fungus *P. hordei* grown on plant tissue agar such as macerated tulip and yellow onion, oatmeal and red onion, while it was not detected in CYA (caffeic acid agar), MEA (malt extract agar), and YES (yeast extract with supplements) media (Overy et al., 2005). When cultivated in rice medium, a hard coral-derived fungus *Scopulariopsis* sp. from the coastline of Red Sea was shown to afford six secondary metabolites including xanthone derivatives (**39**–**40**), phenolic bisabolane-type sesquiterpenes (**41**–**42**), one new alkaloid (**43**) and one new α-pyrone derivative (**44**) (Elnaggar et al., 2016). Interestingly, this strain could biosynthesize a new naphthoquinone derivative (**45**) and two new triterpenoids (**46**–**47**) in the protein-rich white bean medium (Elnaggar et al., 2017).

Chemical investigation of one marine-derived strain *Streptomyces* sp. C34 grown on ISP2 (yeast malt extract agar) medium led to the isolation of four new ansamycin-type polyketides (**48**–**49**). But only compounds **48, 50**, and **51** could be extracted from modified ISP2 medium, which contained glycerol rather than glucose. Bioassay results indicated that


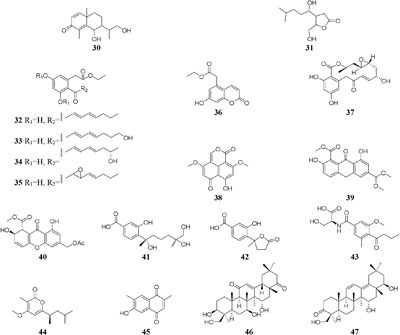



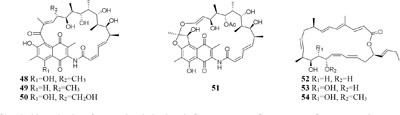


compound **51** had a selective inhibitory effect on *S. aureus* ATCC 25923 with a MIC value of 0.05 μg/mL (Rateb et al., 2011a). The utilization of a defined medium to cultivate strain C34 resulted in the observation of three novel 22-membered lactone polyketides (**52**–**54**) (Reid et al., 1995). Compounds **50**–**52** possessed strong antibacterial activities against *L. monocytogenes* and *B. subtilis* with MIC values range from 3 to 6 μg/mL and against *S. aureus* with MIC values of <1 μg/mL (Rateb et al., 2011a). Four media applied to strain *Streptomyces* sp. CS resulted in production of various natural products including three new macrolides (**55–57**) from YMG agar medium, five new 16-membered macrolides (**58–62**) from ISP2 broth, five novel polyketides (**63**–**67**) from sterilized Waksman Synthetic medium and three new naphthomycins (**68–70**) from oatmeal medium. Compounds **55** was shown to have inhibitory effect on *Fusarium moniliforme* with a MIC value of 300 μg/mL and compounds **58–62** exhibited cytotoxicity toward the MDA-MB-435 human cancer cell line with IC_50_ values of 4.2, 4.5, 5.5, 3.8, and 11.4 mM, respectively (Lu and Shen, 2003, 2004; Li et al., 2008, 2010; Yang et al., 2012). *Streptomyces* sp. ML55 in a medium consisting of glycerin, molasses, casein, polypeptone led to the isolation of three novel antimycins, JBIR-02 (**71**), JBIR-06 (**72**), and JBIR-52 (**73**), while this strain had capacity to produce two novel depsipeptides (**74–75**) in GYM medium (Ueda et al., 2007, 2008; Kozone et al., 2009; Li X. et al., 2013). An ant-derived actinomycete *Streptomyces* sp. 1H-GS5 was found to produce one new spectinabilin derivative (**76**) when cultivated in the medium consisting of corn starch 10%, soybean powder 1%, cotton flour 1%, α-amylase 0.02%, NaCl 0.1%, K_2_HPO_4_ 0.2%, MgSO4 ⋅ 7H_2_O 0.1%, CaCO_3_ 0.7%, cyclohexanecarboxylic acid 0.1%, pH 7.0, while this stain made another new cytotoxic spectinabilin (**77**) when reducing the proportion of nutrients (Liu S. et al., 2015; Liu C. X. et al., 2016).


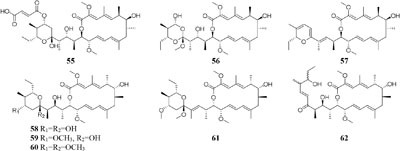



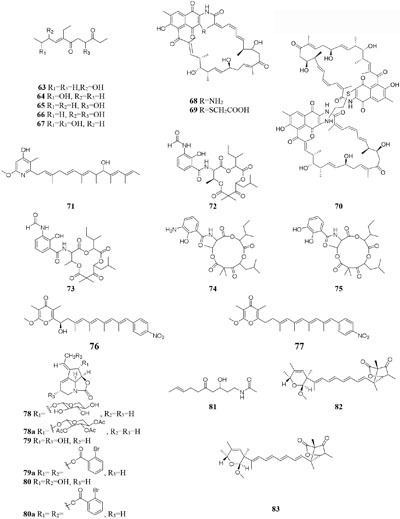



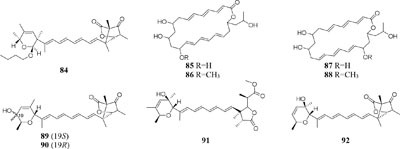


When cultured in an oat bran medium, one strain *Streptomyces* sp. A1 was found to produce rubromycin derivatives, while other three known compounds were biosynthesized in a mannitol/soybean meal medium and three new congeners (**78**–**80**) and streptenol E (**81**) in medium (degreased soybean meal 2%, mannitol 2%, agar 2%) with soil as an addition, provide. Compound **81** had significant cytostatic effect on four tumor cell lines including HMO2, HEP G2, MCF7 and Kato III with GI_50_ values (the concentration that causes 50% growth inhibition) of 0.15, 0.3, 10, and 0.7 μM, respectively (Puder et al., 2001). Phytochemical study of one filamentous soil fungus, *Talaromyces wortmannii*, cultivated in maize culture medium, led to the separation of three new polyketones (**82**–**84**), which were absent in rice or dextrose agar media. Compounds **82**–**84** displayed inhibitory activities against NFRD (fumarate reductase) with IC_50_ values of 8.8, 11, and 13 μM, respectively (Liu W. C. et al., 2016). Interestingly, this stain was found to produce four novel 22-membered macrolides (**85**–**88**) (Dong et al., 2006) and four novel tetraene lactones (**89**–**92**) (Dong et al., 2009) when grown in the still-cultured medium (2.5% soybean meal and 97.5% rice). Compounds **85**–**88** exhibited *in vitro* moderate cytotoxic activities against human cancer cell lines (HCT-5, HCT115, A549, MDA-MB-231, and K562) with IC_50_ values range from 28.7 to 130.5 μM, while compounds **89**–**92** showed potent inhibitory effects on cathepsin B.

### Salinity

Salinity is an important factor in determining many aspects of the chemistry of natural water and of biochemical process within cultivation system, and is a thermodynamic state variable that, along with temperature and pressure, governs physical characteristics like the osmotic pressure and enzymes involved in microbial growth and metabolism (Blunt et al., 2015). Suitable salinity is needed for normal microbial growth and high osmotic pressure makes cells dehydrated and affects microbial biochemical reactions (Poolman and Glaasker, 1998; Wang Y. et al., 2011).

Microorganisms exposed to different types of media supplemented with various halogens maybe trigger their synthesis pathway to restore osmotic imbalance, thus activating different hidden MSM biosynthetic gene clusters. Compare to that grown in seawater, one marine-derived fungus *Aspergillus unguis* CRI282-03 was shown to produce new brominated depsidones (**93**–**95**) and two new orcinol derivatives (**96**–**97**) in KBr medium and a new depsidone (**98**) in KI broth (Sureram et al., 2013). Bioassay results indicated that compounds **95** and **96** possesses aromatase inhibitory effects (Sureram et al., 2012). Nine new polyketides (**99**–**107**), which were absent in the broth contained KI or deionized water, were produced by the fungus *Dothideomycete* sp. CRI7 isolated from *Tiliacora triandra* when cultivated in the medium supplemented with KBr and seawater (Wijesekera et al., 2017).

Chemical investigation of one symbiotic stain *Aspergillus* sp. D from *Edgeworthia chrysantha* led to isolation of five known heterocyclic alkaloids from normal Czapek medium, while a new meroterpenoid (**108**) and four known analogs were obtained from Czapek medium with 3% salty (Zhang et al., 2018a,b). One mangrove-derived endophyte *Wallemia sebi* PXP-89 cultivated in 10% NaCl broth produced a new cyclopentanol pyridine alkaloid (**109**), which was not detected in normal medium (Peng et al., 2011). When cultivated in medium containing 10% sea salt, strain *Spicaria elegans* KLA-03 was shown to biosynthesize a new antimicrobial diacrylic acid (**110**) (Wang F. Z. et al., 2011). Strain *Streptomyces* sp. DSM 14386 could metabolize five new compounds (**111**–**115**) in 1.5% NaCl medium, while this strain produced two brominated congeners (**116–117**) in 1.5% NaBr medium. Antimicrobial tests showed that compounds **113** and **117** displayed potent antibiotics against MRSA (methicillin-resistant *Staphylococcus aureus*) with the same MIC values of 16 μg/mL, and compound **117** also had strong activity toward *Mycobacterium smegmatis* (IC_80_ = 2 μg/mL) (Onaka, 2017). Two rare epidithiodiketopiperazines, gliovirin and pretrichodermamide A, were detected in 1.5% NaCl broth of a marine-derived *Trichoderma* sp. TPU199, while this strain produced a new iodo derivative (**118**) from freshwater medium with 3.0% NaI and 3.0% NaBr as well as 5-bromo-5-deoxy derivative (Yamazaki et al., 2015a).


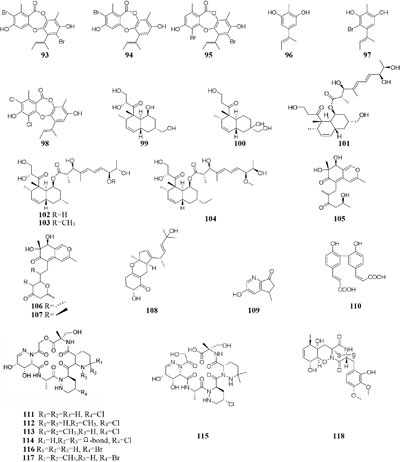


### Metal Ion

Metal ion affects physiological structure and function of microorganism. The interaction between metal ion and microbe is usually assumed in three pathways, including causing reactions in cells, conserving energy in the process of dissimilation, and assimilating reactions (Thorneley, 1990).

One marine-derived strain *Ascotricha* sp. ZJ-M-5 was shown to produce a new 3,4-split ring lanolin alkyl triterpene (**119**) and a new cyclonerols derivative (**120**), when cultivated in eutrophic medium made up with sea salt (Xie et al., 2013a,b). However, three new caryophyllene derivatives (**121**–**123**) were detected in modified Czapek Dox medium, while compound **122** was absent in the fermentation broth without Mg^2+^ (Wang W.J. et al., 2014). Strain *Aspergillus sclerotiorum* C10WU derived from hydrothermal vent sediment in Taiwan (China) could produce three new alkaloids (**124**–**126**) under normal medium. However, this strain metabolized one unelucidated compound due to the low amount available together with aspochracin when grown in the stressed culture medium with Cu^2+^ as a supplement. Likewise, two compounds, namely deoxytryptoquivaline and tryptoquivaline A (**127**–**128**), were purified from the normal extract of *A. clavatus* C2WU, while only metabolite **129** was found in normal medium containing Cu^2+^ and Cd^3+^ (Jiang et al., 2014). A novel antibacterial cyclodepsipeptide, named NC-1 (**130**), was produced by a red soil-derived strain *Streptomyces* sp. FXJ1.172 when cultured in GYM (glucose-yeast extract-malt extract) medium added with ferric ion (Liu M. et al., 2016).


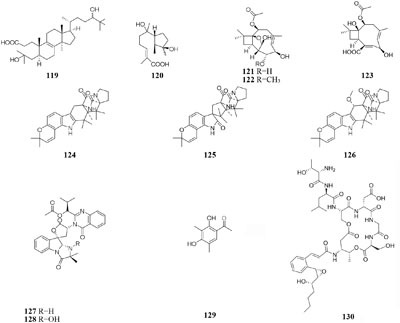


## Cultivation Condition

Suitable cultivation conditions, such as appropriate temperature, pH, oxygen concentration, and cultivation status, are essential for the growth and biochemical reactions of microorganisms. However, many biosynthetic genes of MSMs are not expressed under normal culture conditions, thus it is essential to change the cultivation condition to activate these silent gene clusters to diversify their MSMs.

### Temperature

Chemical diversity of MSM is directly influenced by microbe enzyme activity, which is susceptible to cultivation temperature. The normal function of microbial enzyme is dependent on appropriate temperature. Generally, the higher the cultivation temperature is, the faster the enzyme deactivation rate will be (Feller et al., 1994). For example, when the temperature was lower than 30°C, secondary metabolites of an uncoded strain *Streptomyces* sp. were composed of chlortetracycline, while only tetracycline was synthesized when cultivation temperature went up to 35°C (Cui et al., 1996).

### pH

During microbe fermentation process, the decomposition and utilization of nutrients as well as the accumulation of secondary metabolites usually causes the variation of medium pH (Gibson et al., 1988; Tan et al., 1998). It affects not only the activity of each enzyme, but also the surface charge of the membrane. The nature and permeability of cell membrane could change the rate of utilization of substrate, thus affecting the growth of microorganisms and biosynthesis of final products. Chemical study of one desert-derived strain *Nocardiopsis alkaliphila* nov. YIM-80379 led to isolation of two new pyran-2-one derivatives (**131**–**132**) when cultivated on Gause’s synthetic agar slants with pH = 10. However, the neutral medium was unsuitable for its growth (Hozzein et al., 2004; Wang et al., 2013c). Acidic medium (pH = 5) dramatically increased the production of bioactive compounds of a mangrove-derived fungus *Rhytidhysteron rufulum* AS21B, including two new antitumor spirobisnaphthalenes (**133**–**134**). However, these compounds were not detected in neutral medium (Siridechakorn et al., 2017).

### Oxygen Concentration

Changes in oxygen supply can affect the biochemical reactions and activate different set of functional gene clusters for different secondary metabolites production (Sato, 1990). For example, [^13^C]-labeled acetates and a small amount of [^18^O_2_] were used to investigate the biosynthetic pathway of aspinonene (**135**) in the culture broth of *Aspergillus ochraceus* DSM-7428. It is interesting that aspyrone (**136**) was produced by increasing dissolved oxygen concentration during fermentation, accompanied by reduced amounts of compound **135** under an oxygen enriched atmosphere (Fuchser et al., 1995).


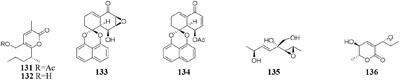


### Cultivation Status

A growing body of evidence has indicated that cultivation status can directly affect microbe metabolic process, including solid or liquid, static or dynamic. Compared with solid and static cultivation, liquid and dynamic modes not only ensure the full contact of microorganisms and nutrients, but also affect their biochemical reactions by changing oxygen supply and activating functional gene clusters. Till now, MSMs from 12 genera had been investigated under different fermentation status, including *Arthrinium, Aspergillus, Myxotrichum, Nodulisporium, Lentinus, Paraphaeosphaeria, Penicillium, Pestalotiopsis, Phomopsis, Spicaria, Streptomyces, Ulocladium*.

#### Arthrinium

One marine sponge-derived fungus *Arthrinium arundinis* ZSDS1-F was shown to metabolize a novel naphthalene glycoside (**137**) (Wang J.F. et al., 2014), five cytochalasins (**138**–**142**) (Wang et al., 2015b), and three alkaloids (**143**–**145**) when cultivated in a rotary liquid medium (Wang et al., 2015c). However, only phenethyl 5-hydroxy-4-oxohexanoate (**146**) was traced in rice medium (Li Y. L. et al., 2017). Bioassay suggested that compounds **143**–**146** possessed *in vitro* cytotoxicity against cancer cell lines A549, BGC823, Huh-7, K562, H1975, MCF-7, HL60, U937, Hela, and MOLT-4 with IC_50_ values in range of 0.24–45 μM. In addition, compounds **143** and **145** displayed significant AchE (acetylcholine esterase) inhibitory activity with IC_50_ values of 47 and 0.81 μM, respectively.


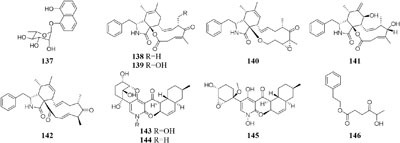


#### Aspergillus

By comparison of solid and liquid fermentation products of an endophytic strain *A. fumigates* LN-4 from stem bark of *Melia azedarach* L., their HPLC profiles were obviously different (Zhang et al., 2013). Strain *A. versicolor* ZLN-60 could produce two new cyclic pentapeptides (**147**–**148**) and four new prenylated diphenyl ethers (**149**–**152**) in static liquid condition (Zhou et al., 2011; Gao et al., 2013). Biological tests indicated that compound **151** displayed moderate cytotoxicity against Hela and K562 cancer cell lines with IC_50_ values of 31.5, 48.9 μM, respectively. However, further purification of its crude extract of solid medium resulted in the detection of four other novel cyclic peptides (**153**–**157**) (Peng et al., 2014). Chemical study of one marine-derived fungus *A. terreus* cultivated in 11 different culture conditions indicated that static agar was ideal for the production of antifungal lovastatins (**158**–**159**) and 7-desmethylcitreoviridin (**160**), which were absent in the shaking fermentation (Adpressa and Loesgen, 2016).


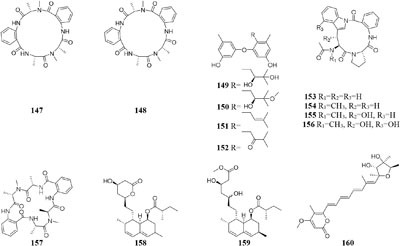


#### Lentinus

Two new prenyl phenols (**161–162**), one indole alkaloid echinuline (**163**) and one anthraquinone fiscione (**164**), were biosynthesized by *Lentinus strigellus* under static condition. While in shaking fermentation broth, this strain produced benzopyrans (**165**–**168)** together with panepoxydone (**169**) and isopanepoxydone (**170**). Bioassay indicated that striguellone A (**171**) displayed moderate cytotoxicity against HeLa cancer cells (Zheng et al., 2009; Barros et al., 2012).


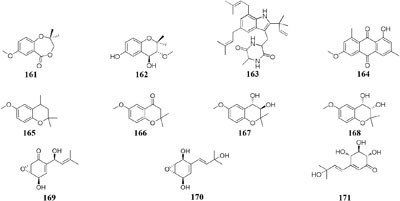


#### Myxotrichum

One fungal strain *Myxotrichum* sp. isolated from lichen *Cetraria islandica* (L.) Ach in Laojun Mountain (China), was shown to make one novel austdiol analog (**172**), three new fulvic acid derivatives (**173**–**175**) and a new citromycetin analog (**176**) in rotary PDB medium (Yuan et al., 2013), while four new polyketides (**177**–**180**) were acquired from rice medium under static fermentation status. And compound **179** was shown to restrain Arabidopsis seeds root markedly with the inhibition rate of 75.9% at 8 μg/mL (Yuan et al., 2016).

#### Nodulisporium

Chemical investigation of one symbiotic strain *Nodulisporium* sp. (No. 65-12-7-1) from the lichen *Everniastrum* sp. resulted in the isolation of two rarely 4-methyl-progesteroids (**181**–**182**) when grown in rice medium (Zheng et al., 2013). Whereas this strain could biosynthesize ten novel nodulisporisteroids (**183**–**192**) in shaking PDB medium (Zhao et al., 2015).


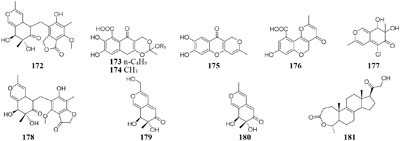



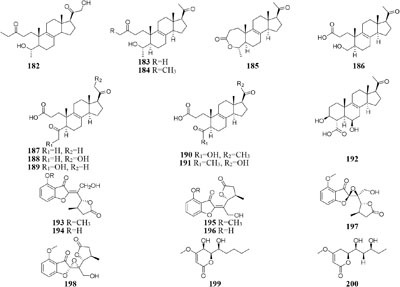


#### Paraphaeosphaeria

A fungal strain *Paraphaeosphaeria photiniae*, inhabiting *Roystonea regia* collected from Jianfeng Mountain (China), was shown to yield six new unique benzofuranone-derived *γ*-lactones (**193**–**198**) when cultivated in shaking liquid medium (Ding et al., 2009), while only two different *δ*-lactone derivatives (**199**–**200**) were detected in its rice medium (Ding et al., 2012).

#### Penicillium

When grown on solid PDA medium, one mangrove-derived fungus *Penicillium brocae* MA-231 could produce six new disulfide-bridged diketopiperazine derivatives (**201**–**206**). Bioassay results showed that compounds **201, 202, 205**, and **206** had cytotoxic activities against Du145, Hela, HepG2, MCF-7, NCI-H460, SGC-7901, SW1990, SW480, and U251 tumor cell lines with IC_50_ values ranging from 0.89 to 9.0 μM (Meng et al., 2014). When cultivated in liquid media (PDB or Czapek), however, five new penicibrocazines (**207**–**211**), four new thiodiketopiperazine alkaloids (**212**–**215**) and two new *N*-containing *p*-hydroxyphenopyrrozin derivatives (**216**–**217**) were detected in its fresh mycelia, which compounds **207**–**209** displayed antimicrobial activities against *Staphylococcus aureus* with MIC values of 32.0, 0.25, 8.0 μg/mL, respectively. In addition, **209**–**211** exhibited potent antimicrobial effect on *Gaeumannomyces graminis* with MIC values of 0.25, 8.0 and 0.25 μg/mL, respectively. And compound **216** showed powerful inhibitory effect on *Fusarium oxysporum* and *S. aureus* (Meng et al., 2015b, 2017).


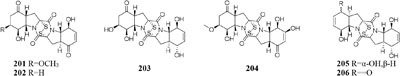



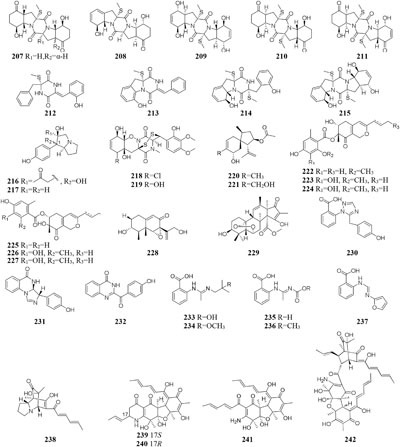


Chemical study of one marine sponge-derived strain *P. adametzioides* AS-53 led to isolation of two new bisthiodiketopiperazine derivatives (**218**–**219**) from shaking PDB broth, whereas two new acorane sesquiterpenes (**220**–**221**) were found in its static rice medium. Compound **218** showed strong lethality against brine shrimp (*Artemia salina*) with an LD_50_ value of 4.8 μM and a broad spectrum of antimicrobial effect on *Aeromonas hydrophilia, S. aureus, Vibrio* spp. *V. harveyi, Gaeumannomyces graminis* and *V. parahaemolyticus* (Liu Y. et al., 2015). Six novel azaphilone derivatives (**222**–**227**) as major secondary metabolites were obtained from rotary PDB medium of one marine-derived strain *P. commune* QSD-17 (Gao et al., 2011), whereas other new compounds isophomenone (**228**) and 3-deacetylcitreohybridonol (**229**) were detected in its static rice medium (Gao et al., 2012).

Three novel penipanoids (**230**–**232**) were characterized from one marine-derived strain *P. paneum* SD-44 grown in rice medium (Li et al., 2011). The exploration of changing fermentation conditions of *P. paneum* SD-44 to a seawater-based culture broth under dynamic fermentation condition gave five new anthranilic acid derivatives (**233**–**237**). Metabolites **233** and **237** exhibited inhibitory activity toward human colon cancer RKO cell lines with IC_50_ values of 8.4, 9.7 μM, respectively (Li C. S. et al., 2013). One deep sea-derived fungus *Penicillium* sp. F23-2 biosynthesized terpenoids, diketopiperazines, and meleagrin alkaloids when incubated in sea-water-based culture medium under static condition (Du et al., 2009, 2010), whereas five new nitrogen-containing sorbicillinoids (**238**–**242**) were metabolized by this stain when cultivated in PYG (peptone yeast glucose) medium under shaking status (Guo et al., 2013).

#### Pestalotiopsis

When grown in rice medium, one endophytic strain of *Pestalotiopsis fici* from *Camellia sinensis* was found to be a prolific producer of bioactive secondary metabolites, including pupukeanane chloride (**243**) (Liu et al., 2008a), chloropestolide A (**244**) (Liu et al., 2009a), seven isoprenylated chromones (**245**–**251**) (Liu et al., 2010), three highly functionalized compounds (**252**–**254**) (Liu et al., 2009b), and three cytotoxic pupukeanane chlorides (**255**–**257**) (Liu et al., 2011). *In vitro* cytotoxic assays suggested that compound **244** possessed potent inhibitory effects on HeLa and HT29 with GI_50_ values of 0.7, 4.2 μM, respectively. However, this strain produced new cyclopropane derivatives (**258**–**262**) when cultivated in shaking liquid medium (Liu et al., 2008b). An endophytic fungus *P. foedan*, residing in *Bruguiera sexangul*, synthesized a new reduced spiro azaphilone derivative (**263**) together with two new isobenzofuranones (**264**–**265**) in solid GYM (glucose, yeast extract, malt) medium (Ding et al., 2008). But, in liquid modified PDB medium, a pair of novel spiro-*γ*-lactone enantiomers (**266**–**267**) were identified (Yang and Li, 2013).


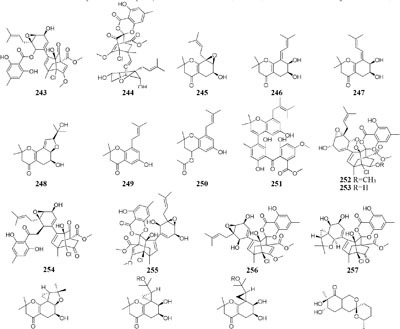



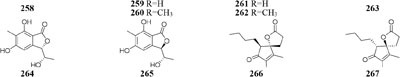


#### Phomopsis

An endophytic fungus *Phomopsis* sp. sh917 isolated from fresh stems of *Isodon eriocalyx var. laxiflora* collected in Kunming Botanical Garden of China, was shown to produce six new polyketides (**268**–**273**) on solid rice medium but metabolize a new polyketide (**274**) in shaking liquid FM4 medium (Tang et al., 2017).

#### Spicaria

Nine new cytochalasins Z7-Z15 (**275**–**283**), one novel spicochalasin (**284**), five new aspochalasins (**285**–**289**), and three new aspochalasin derivatives (**290**–**292**) were synthesized by a marine-derived fungus *Spicaria elegans* KLA03 in the seawater-based medium under static fermentation status. Compounds **235** and **276** displayed strong cytotoxicity against P388 and A-549 cancer cell lines with IC_50_ values in range of 8.4–99 μM (Liu et al., 2005, 2006, 2008c; Lin et al., 2009a, 2010). However, new aromatic polyketide (**293**) was obtained from shaking seawater medium (Luan et al., 2014).


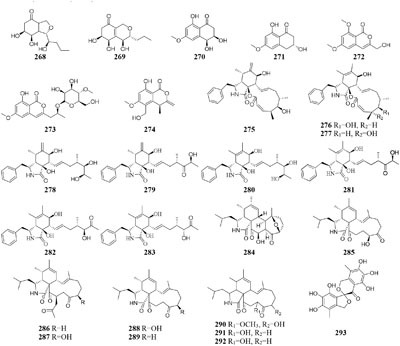


#### Streptomyces

One marine-derived stain *Streptomyces* sp. CHQ-64 was found to produce six new antifungal polyene-polyols (**294–299**) and two new cytotoxic hybrid isoprenoid alkaloids (**300–301**) in liquid medium under shaking condition, while this strain made only one new hybrid isoprenoid alkaloid (**302**) under static condition (Che et al., 2012, 2013, 2015, 2016). When cultivated in liquid Gause’s No. 1 medium, strain *Streptomyces* sp. DT-A37 could produce a new ring-opened lactam (**303**), while in rice medium one unknown holomycin (**304**) and two new cyclopropaneacetic acids (**305**–**306)** were detected (Ding et al., 2017). Strain *Streptomyces* sp. HZP-2216E cultured in 2216E solid medium, GYM solid medium and GMSS (Gause’s medium with sea salt) liquid medium resulted in isolation of two new compounds of 23-*O*-butyrylbafilomycin D (**307**), streptoarylpyrazinone A (**308**) a unique indolizinium alkaloid streptopertusacin A (**309**). It was noted that compound **307** showed potent activity in suppressing the proliferation of the four tested glioma cell lines with IC_50_ values in a range from 0.35 to 2.95 μM and antibacterial activity with MIC value of 7.4 μM for MRSA and IC_50_ values of 0.44 to 0.98 μM for glioma cells (Zhang et al., 2017c,d).

#### Ulocladium

Two antifungal polyketides (**310**–**311**) were characterized from rice medium of *Ulocladium* sp. that was isolated from the lichen *Everniastrum* sp. (Wang X.E. et al., 2012), whereas three new tricycloalternarenes F-H (**312**–**314**) and five ophiobolane sesterterpenes (**315**–**319**) were detected in liquid Czapek or PDB medium (Wang et al., 2013a,b). Compounds **315** and **319** exhibited moderate antibacterial activity against meticillin-resistant *S. aureus* and *Bacillus subtilis* and displayed strong *in vitro* cytotoxicity against cancer cell lines KB and HepG2.


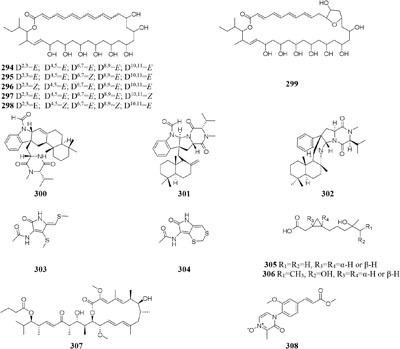



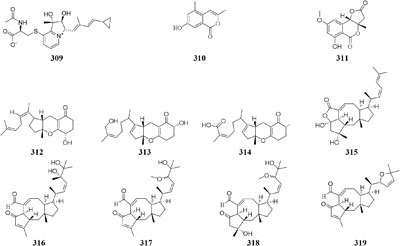


## Co-Cultivation With Other Strain(S)

In one culture medium, the relationship between one strain and other(s) may be competitive, antagonistic or friendly. Co-cultivation of two or more strains usually has positive effect of an enhanced production of known compounds or an accumulation of cryptic compounds that are not detected in axenic culture (Bohni et al., 2013; Marmann et al., 2014). This effect maybe results from the production of enzymes that activate metabolite precursors or that other strain(s) may induce epigenetic modifications of the producer strain.

### Fungus and Other Fungal Strain

An endophytic strain *Acremonium* sp. Tbp-5 from the European yew (*Taxus baccata* L.) could produce new lipoaminopeptides (**320**–**322**) when co-cultivated with *Mycogone rosea* DSM 12973 (Degenkolb et al., 2002). Chemical investigation of the mixed fermentation broth of two epiphytic strains *Aspergillus* sp. FSY-01 and FSW-02 from marine mangrove *Avicennia marina* led to the isolation of a novel alkaloid (**323**), which had antibacterial activity against *Bacillus dysenteriae, B. proteus*, and *E. coli* (Zhu et al., 2011). The production of 2-alkenyl-tetrahydropyran analogs (**224**–**326**) was provoked by *Chaunopycnis* sp. CMB-MF028 in the mixed culture with a partner strain *Trichoderma hamatum* CMB-MF030, which were isolated from the inner tissue of marine pulmonate false limpet (Shang et al., 2017). Co-cultivation of *Monascus* sp. J101, used as the producer of *Monascus* pigment, with *Saccharomyces cerevisiae* KCCM 11371 or *A. oryzae* KCCM 11773 on the solid sucrose medium could result in two folds of accelerated cell growth and 30–40 folds of increased pigment production (Shin et al., 1998). Strain J101 was shown to stimulate cell growth and reproduction by interacting with *S. cerevisiae*, which resulted in production of more hydrophobic pigments compared to the mono-culture (Suh and Shin, 2000a,b). When co-cultivated with *Beauveria felina*, one marine-derived *P. citrinum* could biosynthesize two new compounds (**327**–**328**) featuring in a unique tetracyclic framework, whereas neither strain could produce these compounds in axenic medium. Antimicrobial assay showed that compounds **327** and **328** had strong inhibitory effects on human pathogens *S. aureus* and *E. coli* (Meng et al., 2015a). *Penicillium* sp. IO1 derived from mediterranean sponge *Ircinia oros* could produce a new fusarielin analog (**329**). However, co-cultivation of *Penicillium* strains IO1 and IO2 resulted in the accumulation of two known compounds norlichexanthone and monocerin, which were not detected in axenic controls (Chen et al., 2015a). Four new polyketides (**330**–**333**) were detected in a dual culture of the deep-sea-derived fungus *Talaromyces aculeatus* and a mangrove-derived fungus


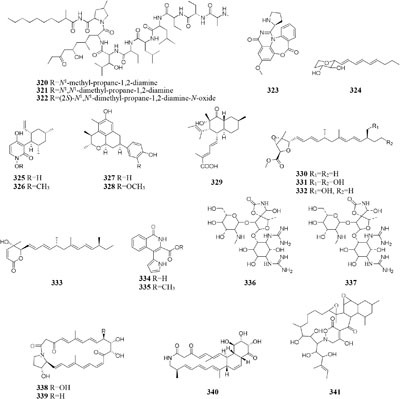


*P. variabile*, while these compounds were not identified in single culture. Compounds **333** displayed strong cytotoxicity against A549, K562, HCT-116, HeLa, MCF-7 and HL-60 human cancer cell lines with IC_50_ values ranging from 1.2 to 9.8 μM (Zhang et al., 2017b).

One novel 1-isoquinolone analog (**334**) and its methyl ester (**335**) were detected in mycelia and culture filtrate of mixed fermentation of two endophytic fungi Nos. 1924^#^ and 3893^#^, whereas these compounds were not traced in axenic medium under the same conditions (Zhu and Lin, 2006). The formation of new antibiotics (**336**–**337**) was emerged during co-cultivation of a multi-antibiotic stable mutant strain of *Rhodococcus fascians* and a strain *Streptomyces padanus*, neither of which was capable of yielding an antibiotic (Kurosawa et al., 2008). An terrestrial bacterium *Tsukamurella pulmonis* TP-B0596 co-cultured with strain *Streptomyces* sp. NZ-6, coincided with stimulation of three new metabolites (**338**–**340**) of unprecedented di-andtricyclic macrolactams (Hoshino et al., 2015). The yield of red pigment was detected in the dual induction of *T. pulmonis* TP-B0596 and *S. lividans* TK23. Co-cultivation of *T. pulmonis* and *S. endus* S-522 resulted in the production of one new antibiotics called alchivemycin A (**341**) (Onaka et al., 2011). A soil-dwelling actinomycetes *S. coelicolor* was shown to significantly improve the yield of red compound undecylprodigiosin, when co-cultured with *Corallococcus coralloides* (Schäberle et al., 2014).

### Bacterium and Other Bacterial Strain

Only two new macrolactams (**342**–**343**) were detected in the co-culture broth of a rare actinomycete *Micromonospora wenchangensis* HEK-797 and *Tsukamurella pulmonis* TPB0596, whereas the axenic medium of strain HEK-797 produced a polyene macrolactam (**344**), which was possibly the precursor of compounds **342** and **343** (Hoshino et al., 2017). Investigation of the interaction of the portable predator *Myxococcus Xanthus* and *Streptomyces coelicolor* showed that actinorhodin production of *S. coelicolor* was raised up to 20-fold and stimulated aerial mycelium production (Pérez et al., 2011). Co-cultivation of two sponge-derived actinomycetes, *Nocardiopsis* sp. RV163 and *Actinokineospora* sp. EG49, induced ten reported compounds, including diketopiperazine, angucycline, and β-carboline derivatives, while only three natural products were isolated in mono-culture (Dashti et al., 2014). Mixed culture of *Pseudomonas maltophilia* 1928 and *S. griseorubiginosus* 43708 resulted in the production of one peptide antibiotic, biphenomycin A (**345**) (Ezaki et al., 1992). However, the accumulation of biphenomycin A, which could be obtained from the transformation of biphenomycin C (**346**), was inhibited in single culture of strain 1928 (Uchida et al., 1985; Ezaki et al., 1993). Interspecies interactions between *Streptomyces coelicolor* M145 with other actinomycete stains (*Amycolatopsis* sp. AA4, *Streptomyces* sp. E14, *Streptomyces* sp., SPB74 and *S. viridochromogenes* DSM 40736) resulted in the production of at least 12 different versions of a molecule called desferrioxamine (Traxler et al., 2013).

### Fungus and Bacterium

Co-cultivation of one fungal strain *A. terreus* with *B. cereus* and *B. subtilis* resulted in the yield of two novel butyrolactones (**347**–**348**), which were absent in single culture medium (Chen et al., 2015b). An endophyte *Chaetomium* sp. from the Cameroonian plant *Sapium Ellipticum* (Euphorbiaceae) was shown to produce two novel shikimic acid analogs (**349–350**) and four new butenolide derivatives (**351–354**) when co-cultivated with *Pseudomonas aeruginosa*, while none of these chemicals was traced in axenic medium (Ancheeva et al., 2017). Strain *Bacillus subtilis* 168 trpC2 was shown to greatly activate the biosynthesis of three novel chemicals (**355–357**) of fungal endophyte *Fusarium tricinctum* during co-culture process. And these compounds were not duplicated in axenic fungal culture (Ola et al., 2013).


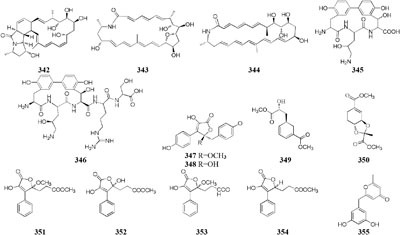



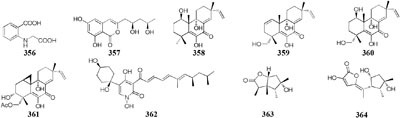


Co-cultivation of one marine fungus *Libertella* sp. CNL-523 symbiotic on an ascidian collected from the Bahamas and a fellow strain *Thalassospira* sp. CNJ-328 resulted in the production of four new diterpenoids (**358**–**361**). Compound **360** exhibited remarkable cytotoxicity against HCT-116 human adenocarcinoma cell line with an IC_50_ value of 0.76 μM (Oh et al., 2005). A new pyridone alkaloid (**362**) was isolated from the mixed culture extract of *Paecilomyces lilacinus* and *Salmonella typhimurium*, which had 57.5 ± 5.50% of AChE inhibition (Teles and Takahashi, 2013). Co-culture of an endophyte *Pestalotiopsis* sp. from *Drepanocarpus lunatus* with *B. subtilis* was found to biosynthesize two novel sesquiterpenoids (**363**–**364**) while new compounds **365** and **366** emerged in axenic culture (Liu et al., 2017). The mixed cultivation of *Trichoderma* sp. 307 colonizing in *Clerodendrum inerme* and one bacterium *Acinetobacter johnsonii* B2 led to the production of two new sesquiterpenes (**367**–**368**) and three novel de-*O*-methyl lasiodiplodins (**369**–**371**). Compounds **369** and **370** displayed potent α-glucosidase inhibitory effect with IC_50_ values of 25.8 and 54.6 μM, respectively (Zhang et al., 2017a).

Chemical study of an endophytic stain *Aspergillus austroafricanus* from *Eichhornia crassipes* led to the isolation of a highly oxygenated heterodimeric xanthone (**372)** and a new sesquiterpene (**373**) in axenic culture. Mixed fermentation of *A. austroafricanus* with *B. subtilis* or *S. lividans* afforded several diphenyl ethers, including one new austramide (**374**) (Ebrahim et al., 2016). Two novel *N*-formyl alkaloids (**375–376**) were characterized from a mixed fermentation of *A. fumigatus* and *S. peucetius*. Compound **376** displayed *in vitro* cytotoxic effect on cancer cell line NCI-60 with an IC_50_ value of 1.12 μM (Zuck et al., 2011). Seven known diketopiperazine alkaloids associated with ergosterol and 11-*O*-methylpseurotin A were traced in response to the supplement of *A. fumigatus* MBC-F1-10 to an established culture of *S. bullii*, whereas neither strain metabolized these compounds when cultivated alone (Rateb et al., 2013). Co-cultivation of *A. fumigatus* MR2012 with *S. leeuwenhoekii* C34 in ISP2 medium resulted in the yield of a new luteoride derivative (**377**) and a new pseurotin derivative (**378**). None of these compounds could be detected in axenic culture. When strain MR2012 was co-cultivated with strain C58, a lasso peptide chaxapeptin (**379**) was made, which displayed significant inhibitory effect on human lung cancer cell line A549 (Elsayed et al., 2015; Wakefield et al., 2017).


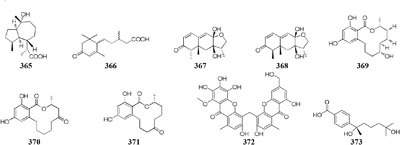



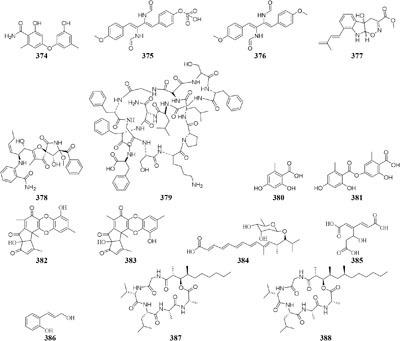


Physical interaction of *A. nidulans* RMS011 with *S. hygroscopicus* was found to trigger biosynthesis of four new aromatic polyketides (**380**–**383**), which were absent in the axenic medium (Schroeckh et al., 2009). A new polyketide glycoside (**384**) was formed in the dual induction of two Gram-positive bacteria, *S. tendae* KMC006 and *Gordonia* sp. KMC005, which were obtained from an acidic mine drainage sample (Park et al., 2017). In response to *S. coelicolor* A3(2) M145, strain *A. niger* N402 was shown to be apt to produce 2-hydroxyphenylacetic acid and cyclic dipeptide cyclo(Phe–Phe). Biotransformation of a new hexadienedioxic acid (**385)** and a new phenol derivative (**386**) was achieved by co-culture of these strains (Wu et al., 2015). More interestingly, co-cultivation of one marine-derived fungus *Emericella* sp. CNL-878 with *Salinispora arenicola* CNH-665 resulted in the higher yields of two novel antimicrobial cyclic depsipeptides (**387**–**388**) than axenic culture (Oh et al., 2007).

## Epigenetic Modifier

Epigenetic modifiers are those chemicals that are able to change microbial characteristics in correspondence to alteration of their epigenetic status, such as DNA methyltransferase (DNMT) inhibitor and histone deacetylase (HDAC) inhibitor. The addition of these modifiers usually suppresses the activity of related enzymes in the biosynthetic pathway and promotes the progress of other metabolic pathways (Seyedsayamdost, 2014).

### DNA Methyltransferase Inhibitor

DNA methylation is a process by which methyl groups are added to DNA. When located in a gene promoter, DNA methylation typically acts to repress gene transcription and causes chromatin structure changes in the corresponding regions, preventing the binding of specific transcription factors and suppressing gene expression (Araujo et al., 2001). 5-Azacytidine (5-AC) is the most common DNMT inhibitor used to modify the function of microbe DNA followed by repressing gene transcription. Chemical investigation of a marine-derived fungus *Aspergillus sydowii* afforded three novel bisabolane-type sesquiterpenoids (**389**–**391**) when its culture medium was supplemented with 5-AC (Chung et al., 2013b). An entomopathogenic fungus *Cordyceps indigotica* yielded a novel aromatic polyketide


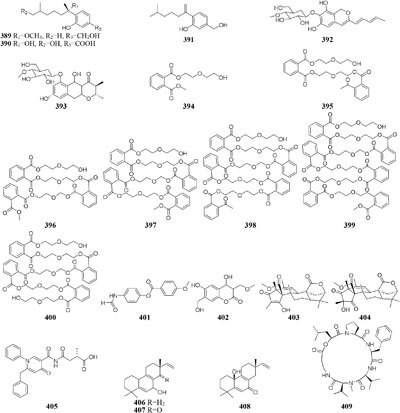



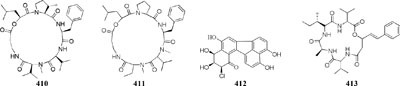


glycoside (**392**) when cultivated in PDB media, while the strain produced another unusual glycoside (**393**) when supplement with 5-AC (Asai et al., 2012e). Several other examples that adding 5-AC as epigenetic modifier in culture medium could lead to the production of new metabolites were also reported, such as novel diethylene glycol phthalate esters (**394**–**400**) from a marine-derived strain *Cochliobolus lunatus* TA26-46 (Chen et al., 2016), a new benzoic acid (**401**) from *Pestalotiopsis microspora* (Yang et al., 2017), one new coumarin (**402**) from *P. crassiuscula* NBRC 31055 associated with *Fragaria chiloensis* (Yang et al., 2014), and novel meroterpenes (**403**–**404**) from *P. citreonigrum* (Wang et al., 2010).

### Histone Deacetylase Inhibitor

The acetylation or deacetylation of histone affects its binding to DNA in microbe. There are many chemical modifications in the tail of histone that regulate the gene expression. The introduction of hydrophobic acetyl group into the *N*-terminal lysine residues of histone could increase the electrostatic attraction and steric hindrance between histone and DNA, which is conducive to facilitate the depolymerization of DNA and the binding of transcription factors (Fukuda et al., 2006; Cole, 2008). Suberoyl bishydroxamic acid (SBHA), suberoylanilide hydroxamic acid (SAHA), and nicotinamide are the most common HDAC chemicals used to inhibit the deacetylation and facilitate gene transcription and expression in microbes (Moore et al., 2012).

Many reports suggested the presence of SAHA in culture medium could result in production of new natural compounds, such as a novel metabolite nygerone A (**405**) from a soil-dwelling fungus *A. niger* ATCC 1015 (Henrikson et al., 2009), two new aromatic norditerpenes (**406**–**407**) tied with an oxygenated derivative (**408**) from a marine-derived *A. wentii* na-3 residing in the brown alga *Sargassum fusiforme* (Miao et al., 2014), three novel cyclodepsipeptides (**409**–**411**) from *Beauveria feline* (Chung et al., 2013a), one novel chlorinated polyketide (**412**) from *Daldinia* sp. (Du et al., 2014), a new cyclodepsipeptide of hybrid EGM-556 (**413**) from one marine sediment-derived fungus *Microascus* sp. (Vervoort et al., 2011).


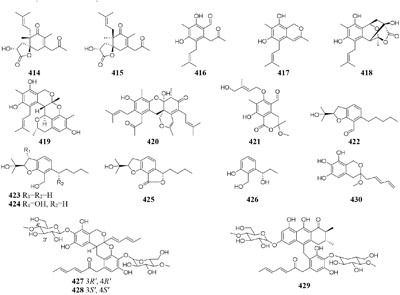



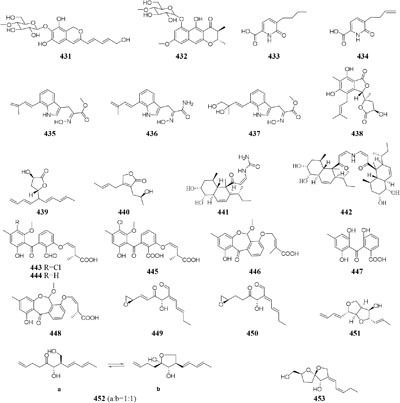


In SBHA-treated culture medium, *Chaetomium indicum* could produce two novel spironolactone polyketides (**414**–**415**) and six novel prenylated aromatic polyketides (**416**–**421**) (Asai et al., 2013b,c). Similarly, when exposed to SBHA, four new 2,3-dihydrobenzofurans (**422–425**) and a new aromatic polyketide (**426**) were characterized from an entomopathogenic fungus *Cordyceps annullata* (Asai et al., 2012c), six new aromatic polyketides (**427**–**432**) were synthesized by *C. indigotica* (Asai et al., 2012f), two new fusaric acid derivatives (**433**–**434**) were produced by *Fusarium oxysporum* associated with medicinal plant *Datura stramonium* L. (Chen et al., 2013), and a series of novel prenylated tryptophan analogs (**435**–**437**) were metabolized by an entomopathogenic fungus *Torrubiella luteorostrata* (Asai et al., 2011). Supplement of nicotinamide [a Zn(II)-type HDAC inhibitor] in culture medium of *C. cancroideum* could generate three novel polyketides (**438**–**440**) (Asai et al., 2016). The use of this inhibitor to strains *Eupenicillium* sp. LG41 and *Graphiopsis chlorocephala* had the similar effect, which the former supplied two new decalin-containing compounds (**441**–**442**) (Li G. et al., 2017) and the later afforded a serious of new benzophenones (**443**–**444**) and diverse new C^13^-polyketides (**445**–**453**) (Asai et al., 2012d, 2013a).

### Multiple Chemical Epigenetic Modifiers

Interactions between epigenetic features play an important role in regulation of gene expressing or silencing in microorganisms, such as DNA methylation and histone modification. Many references that looked into the combined effect of epigenetic processes suggested that these chemicals could regulate the activity of genomic regions of varying sizes, from single genes to entire domains and chromosomes. Epigenetic markers could also interact with other nuclear proteins to work together to form chromatin structures and to create genomic functional discrete regions that induce the production of new secondary metabolites (Tammen et al., 2013).

One symbiotic strain *Alternaria* sp. from medicinal plant *Datura stramonium Linn.* was shown to produce four new aromatic polyketides (**454**–**457**) and a new tenuazonic acid (**458**) when incubated in medium containing 5-AC and/or SBHA. While these compounds were absent in normal culture medium. Interestingly, the yield of these secondary metabolites was higher in the medium of adding HDAC and DNMT inhibitors than that of addition of any other inhibitors (Sun et al., 2012). Chemical investigation of one marine-derived fungus *Aspergillus* sp. SCSIOW2 or SCSIOW3, exposed with an integration of SHBA and 5-AC, led to production of three new eremophilane-type sesquiterpenes (**459**–**461**) together with a new diphenylether-*O*-glycoside (**462**) (Wang L. et al., 2016; Li X. et al., 2017). Bioactivity tests indicated that the glycosylated compound **462** exhibited a protective activity toward free radicals with an EC_50_ value of 20.8 μM. One strain *Cladosporium cladosporioides* from a tidal pool was found to display different responses to the treatment with 5-AC and SHBA. Exposure of *C. cladosporioides* to 5-AC resulted in substantially increased biosynthesis of three oxylipins (**463**–**465**), whereas SHBA induced the yield of two new perylenequinones (**466**–**467**) (Williams et al., 2008).


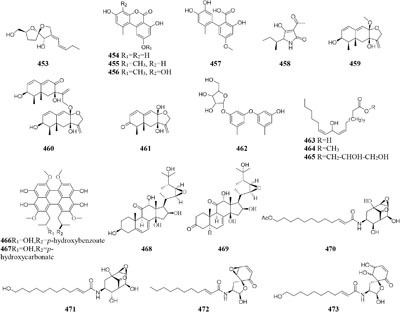



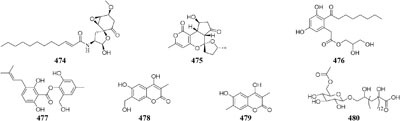


Concomitant supplement of SHBA and *N*-phthalyl-*L*-tryptophan (DNMT inhibitor) to the fermentation medium of an entomopathogenic fungus *Gibellula formosana* induced the formation of two new highly oxidized ergosterols (**468**–**469**) and five new isariotin analogs (**470**–**474**) (Asai et al., 2012a). The same method was applied to expand MSM profile of *Isaria tenuipes*, which resulted in the yield of one new polyketide (**475**) (Asai et al., 2012b). An endophytic strain *Leucostoma persoonii* from red mangrove was subject to large-scale cultivation with sodium butyrate (HDAC inhibitor) and 5-AC, which resulted in the increased yield of known cytosporones and the production of one new cytosporone (**476**) (Beau et al., 2012). Three novel aromatics (**477**–**479**) were produced by *Pestalotiopsis acacia* from *Taxus brevifolia* when its culture medium was supplemented with SHBA and 5-AC (Yang and Li, 2013). Application of this approach also led to production of a new glycolipid ustilagic acid C (**480**) by *Ustilago maydis* (Yang et al., 2013).

## Other Factors

### Enzyme Inhibitor

Beside DNMT and HDAC, other microbial enzymes also played important role in the regulating the biosynthesis of secondary metabolites, such as monooxygenase and hydroxylase. Some chemicals can selectively inhibit the activity of these enzymes in the biosynthetic pathway and promote the progress of other metabolic pathways, such as metyrapone, tricyclazole, jasplakinolide, and DMSO.

Chemical study of *Chaetomium subaffine* in the presence of metyrapone (an inhibitor of cytochrome P-450) led to purification of five new polyketides (**481**–**485**) and two new less oxidized analogs (**486**–**487**) (Oikawa et al., 1992). A soil-derived strain *Phoma* sp. SNF-1778 was shown to yield a new cytochalasin (**488**) when inoculated with metyrapone (Kakeya et al., 1997). When added with the F-actin inhibitor jasplakinolide in culture medium, one marine sponge-derived fungus *Phomopsis asparagi* could afford three unusual cytotoxic compounds, chaetoglobosin-510 (**489**), chaetoglobosin-540 (**490**), and chaetoglobosin-542 (**491**) (Christian et al., 2005). Two novel bisnaphthalene compounds (**492**–**493**) were characterized from *Sphaeropsidales* sp. F-24’707 cultured with tricyclazole, which was shown to inhibited the regular biosynthesis of 1,8-dihydroxynaphthalene (Bode and Zeeck, 2000). Continuous study showed that metyrapone supplementation in the culture of *Spicaria elegans* led to the isolation of two novel 7-deoxy-cytochalasins (**494**–**495**). Compound **494** had weak


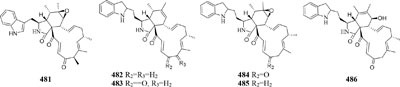



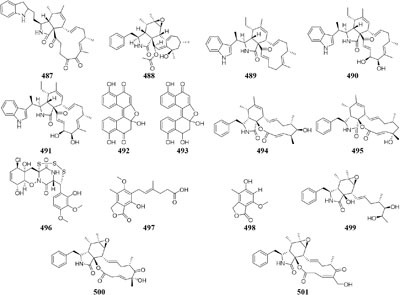


cytotoxicity against human lung cancer cell line A-549 at 15.0 mM (Lin et al., 2009b). One marine-derived strain *Trichoderma* cf. *brevicompactum* elicited an unprecedented epi-diketopiperazine (**496**), which has a trisulfide bond between the α–β positions of two amino acid residues, by adding DMSO to its natural seawater medium (Yamazaki et al., 2015b).

### Biosynthetic Precursor

Biosynthetic precursor refers to one chemical that is apt to be directly incorporated into the final product. Adding various biosynthetic precursors in the fermentation medium may change biosynthesis pathways of secondary metabolites and result in the production of novel compounds (Ramm et al., 2017).

An endophytic strain *Penicillium crustosum* from the ripe berry of *Coffea arabica* L., treated with ferulic acid and quinic acid or cinnamic acid and 3,4-(methylenedioxy) cinnamic acid, was shown to produce mycophenolic acid (**497**) and 5-hydroxy-7-methoxy-4-methylphthalide (**498**) (Valente et al., 2013). Three novel cytochalasins Z_21_–Z_23_ (**499**–**501**) were characterized from one marine-derived fungus *Chaetomium indicum* KLA03 when cultivated in medium supplied by *L*- and *D*-tryptophan. Compound **498** exhibited potent cytotoxic effect on A549 cell lines with an IC_50_ value of 8.2 μM (Wang F. Z. et al., 2011). Strain *S. griseoviridis* Tü 3634 could afford a wide variety of acyl α-*L*-rhamnopyranosides (pyrrolyl, indolyl, thienyl, furanyl, and pyridyl derivatives) if its culture medium, respectively, added corresponding precursors, heteroaromatic carboxylic acid, benzoic acid, cinnamic acid, aminobenzoic acid, and salicylic acid (Grond et al., 2000, 2002).

## Conclusion

Microorganisms are susceptible to culture conditions, such as medium composition, temperature, pH, salinity, culture status, axenic or mixed culture, epigenetic modifier, biosynthetic precursor, and so on. Variation of these factors may result in changing chemical diversity of secondary metabolites. Traditional culture method of microbe is limited to the expression of a large number of metabolic pathways that many MSMs could not be biologically synthesized. A growing body of evidence has suggested that OSMAC strategy can provide a simple, quick and effective approach for enhancing chemo-diversity of MSM to obtain new drug leads through activating silent gene clusters. Moreover, employment of this strategy could avoids the waste of time and resources caused by multiple inoculation, screening, culturing and separation in comparison with mutation strategy (Fang et al., 2014) and ribosome engineering (Ochi et al., 2004). Nowadays, the rate of discovery of new MSM is getting lower and the possibility of the re-discovery of known compounds is higher than before. Therefore, OSMAC strategy would be an important alternative way to alleviate this challenge. There is a great need for new method to assist in isolating and identifying novel bioactive MSMs, such as bioassay-guided isolation, microbe genomes mining (Hug et al., 2018) and LC-MS/MS based molecular networking analysis (Wang M. et al., 2016).

## Author Contributions

RP made a draft of the review. XB and JC searched and collected the references. HZ and HW conceived and revised this review.

## Conflict of Interest Statement

The authors declare that the research was conducted in the absence of any commercial or financial relationships that could be construed as a potential conflict of interest.

## References

[B1] Adpressa D. A. Loesgen S. (2016). Bioprospecting chemical diversity and bioactivity in a marine derived *Aspergillus terreus*. *Chem. Biodivers.* 13 253–259. 10.1002/cbdv.201500310 26880440

[B2] Ancheeva E. Küppers L. Akone S. H. Ebrahim W. Liu Z. Mándi A. (2017). Expanding the metabolic profile of the fungus *Chaetomium* sp. through co-culture with autoclaved *Pseudomonas aeruginosa*. *Eur. J. Org. Chem.* 2017 3256–3264. 10.1002/ejoc.201700288

[B3] Araujo F. D. Croteau S. Slack A. D. Milutinovic S. Bigey P. Price G. B. (2001). The DNMT1 target recognition domain resides in the N terminus. *J. Biol. Chem.* 276 6930–6936. 10.1074/jbc.M009037200 11104769

[B4] Asai T. Chung Y. M. Sakurai H. Ozeki T. Chang F. R. Wu Y. C. (2012a). Highly oxidized ergosterols and isariotin analogs from an entomopathogenic fungus, *Gibellula formosana*, cultivated in the presence of epigenetic modifying agents. *Tetrahedron* 68 5817–5823. 10.1016/j.tet.2012.05.020

[B5] Asai T. Chung Y. M. Sakurai H. Ozeki T. Chang F. R. Yamashita K. (2012b). Tenuipyrone, a novel skeletal polyketide from the entomopathogenic fungus, *Isaria tenuipes*, cultivated in the presence of epigenetic modifiers. *Org. Lett.* 14 513–515. 10.1021/ol203097b 22201477

[B6] Asai T. Luo D. Obara Y. Taniguchi T. Monde K. Yamashita K. (2012c). Dihydrobenzofurans as cannabinoid receptor ligands from *Cordyceps annullata*, an entomopathogenic fungus cultivated in the presence of an HDAC inhibitor. *Tetrahedron Lett.* 53 2239–2243. 10.1016/j.tetlet.2012.02.088

[B7] Asai T. Morita S. Shirata N. Taniguchi T. Monde K. Sakurai H. (2012d). Structural diversity of new C-13-polyketides produced by *Chaetomium mollipilium* cultivated in the presence of a NAD(+)-dependent histone deacetylase inhibitor. *Org. Lett.* 14 5456–5459. 10.1021/ol302539s 23083076

[B8] Asai T. Yamamoto T. Chung Y. M. Chang F. R. Wu Y. C. Yamashita K. (2012e). Aromatic polyketide glycosides from an entomopathogenic fungus, *Cordyceps indigotica*. *Tetrahedron Lett.* 53 277–280. 10.1016/j.tetlet.2011.10.013

[B9] Asai T. Yamamoto T. Oshima Y. (2012f). Aromatic polyketide production in *Cordyceps indigotica*, an entomopathogenic fungus, induced by exposure to a histone deacetylase inhibitor. *Org. Lett.* 14 2006–2009. 10.1021/ol3005062 22480311

[B10] Asai T. Morita S. Taniguchi T. Monde K. Oshima Y. (2016). Epigenetic stimulation of polyketide production in *Chaetomium cancroideum* by an NAD(+)-dependent HDAC inhibitor. *Org. Biomol. Chem.* 14 646–651. 10.1039/c5ob01595b 26549741

[B11] Asai T. Otsuki S. Sakurai H. Yamashita K. Ozeki T. Oshima Y. (2013a). Benzophenones from an endophytic fungus, *Graphiopsis chlorocephala*, from *Paeonia lactiflora* cultivated in the presence of an NAD(+)-dependent HDAC inhibitor. *Org. Lett.* 15 2058–2061. 10.1021/ol400781b 23578108

[B12] Asai T. Taniguchi T. Yamamoto T. Monde K. Oshima Y. (2013b). Structures of spiroindicumides A and B, unprecedented carbon skeletal spirolactones, and determination of the absolute configuration by vibrational circular dichroism exciton approach. *Org. Lett.* 15 4320–4323. 10.1021/ol401741z 23972233

[B13] Asai T. Yamamoto T. Shirata N. Taniguchi T. Monde K. Fujii I. (2013c). Structurally diverse chaetophenol productions induced by chemically mediated epigenetic manipulation of fungal gene expression. *Org. Lett.* 15 3346–3349. 10.1021/ol401386w 23767797

[B14] Asai T. Yamamoto T. Oshima Y. (2011). Histone deacetylase inhibitor induced the production of three novel prenylated tryptophan analogs in the entomopathogenic fungus, *Torrubiella luteorostrata*. *Tetrahedron Lett.* 52 7042–7045. 10.1016/j.tetlet.2011.10.020

[B15] Barros B. A. de Oliveira M. C. F. Mafezoli J. Barbosa F. G. Rodrigues E. (2012). Secondary metabolite production by the basidiomycete, *Lentinus strigellus*, under different culture conditions. *Nat. Prod. Commun.* 7 771–773. 22816304

[B16] Beau J. Mahid N. Burda W. N. Harrington L. Shaw L. N. Mutka T. (2012). Epigenetic tailoring for the production of anti-infective cytosporones from the marine fungus *Leucostoma persoonii*. *Mar. Drugs* 10 762–774. 10.3390/md10040762 22690142 PMC3366674

[B17] Blunt J. W. Copp B. R. Keyzers R. A. Munro M. H. G. Prinsep M. R. (2015). Marine natural products. *Nat. Prod. Rep.* 32 116–211. 10.1039/c4np00144c 25620233

[B18] Bode H. B. Bethe B. Hofs R. Zeeck A. (2002). Big effects from small changes: possible ways to explore nature’s chemical diversity. *Chembiochemistry* 3 619–627. 10.1002/1439-7633(20020703)3:7<619::AID-CBIC619>3.0.CO;2-9 12324995

[B19] Bode H. B. Zeeck A. (2000). Sphaerolone and dihydrosphaerolone, two bisnaphthyl-pigments from the fungus *Sphaeropsidales* sp. F-24 ’ 707. *Phytochemisty* 54 597–601. 10.1016/s0031-9422(00)00145-x 10963453

[B20] Bohni N. Cordero-Maldonado M. L. Maes J. Siverio-Mota D. Marcourt L. Munck S. (2013). Integration of microfractionation, qNMR and zebrafish screening for the *in vivo* bioassay-guided isolation and quantitative bioactivity analysis of natural products. *PLoS One* 8:e64006. 10.1371/journal.pone.0064006 23700445 PMC3660303

[B21] Brzonkalik K. Hümmer D. Syldatk C. Neumann A. (2012). Influence of pH and carbon to nitrogen ratio on mycotoxin production by *Alternaria alternata* in submerged cultivation. *AMB Express* 2:28. 10.1186/2191-0855-2-28 22608165 PMC3441619

[B22] Che Q. Li J. Li D. Gu Q. Zhu T. (2016). Structure and absolute configuration of drimentine I, an alkaloid from *Streptomyces* sp. CHQ-64. *J. Antibiot.* 69 467–469. 10.1038/ja.2015.133 26732256

[B23] Che Q. Li T. Liu X. Yao T. Li J. Gu Q. (2015). Genome scanning inspired isolation of reedsmycins A–F, polyene-polyol macrolides from *Streptomyces* sp. CHQ-64. *RSC Adv.* 5 22777–22782. 10.1039/c4ra15415k

[B24] Che Q. Zhu T. Keyzers R. A. Liu X. Li J. Gu Q. (2013). Polycyclic hybrid isoprenoids from a reed rhizosphere soil derived *Streptomyces* sp. CHQ-64. *J. Nat. Prod.* 76 759–763. 10.1021/np3008864 23547884

[B25] Che Q. Zhu T. Qi X. Mándi A. Kurtán T. Mo X. (2012). Hybrid Isoprenoids from a reeds rhizosphere soil derived actinomycete *Streptomyces* sp. CHQ-64. *Org. Lett.* 14 3438–3441. 10.1021/ol301396h 22702354

[B26] Chen H. Aktas N. Konuklugil B. Mándi A. Daletos G. Lin W. (2015a). A new fusarielin analogue from *Penicillium* sp. isolated from the mediterranean sponge *Ircinia oros*. *Tetrahedron Lett.* 56 5317–5320. 10.1016/j.tetlet.2015.07.072

[B27] Chen H. Daletos G. Abdel-Aziz M. S. Thomy D. Dai H. Brötz-Oesterhelt H. (2015b). Inducing secondary metabolite production by the soil-dwelling fungus *Aspergillus terreus* through bacterial co-culture. *Phytochem. Lett.* 12 35–41. 10.1016/j.phytol.2015.02.009

[B28] Chen H. J. Awakawa T. Sun J. Y. Wakimoto T. Abe I. (2013). Epigenetic modifier-induced biosynthesis of novel fusaric acid derivatives in endophytic fungi from *Datura stramonium* L. *Nat. Prod. Bioprospect.* 3 20–23. 10.1007/s13659-013-0010-2

[B29] Chen M. Zhang W. Shao C. L. Chi Z. M. Wang C. Y. (2016). DNA methyltransferase inhibitor induced fungal biosynthetic products: diethylene glycol phthalate ester oligomers from the marine-derived fungus *Cochliobolus lunatus*. *Mar. Biotechnol.* 18 409–417. 10.1007/s10126-016-9703-y 27245469

[B30] Christian O. E. Compton J. Christian K. R. Mooberry S. L. Valeriote F. A. Crews P. (2005). Using jasplakinolide to turn on pathways that enable the isolation of new chaetoglobosins from *Phomopsis asparagi*. *J. Nat. Prod.* 68 1592–1597. 10.1021/np050293f 16309305 PMC3972004

[B31] Chung Y. M. Elshazly M. Chuang D. W. Hwang T. L. Asai T. Oshima Y. (2013a). Suberoylanilide hydroxamic acid, a histone deacetylase inhibitor, induces the production of anti-inflammatory cyclodepsipeptides from *Beauveria felina*. *J. Nat. Prod.* 76 1260–1266. 10.1021/np400143j 23822585

[B32] Chung Y. M. Wei C. K. Chuang D. W. El-Shazly M. Hsieh C. T. Asai T. (2013b). An epigenetic modifier enhances the production of anti-diabetic and anti-inflammatory sesquiterpenoids from *Aspergillus sydowii*. *Bioorg. Med. Chem.* 21 3866–3872. 10.1016/j.bmc.2013.04.004 23647825

[B33] Cole P. A. (2008). Chemical probes for histone-modifying enzymes. *Nat. Chem. Biol.* 4 590–597. 10.1038/nchembio.111 18800048 PMC2908280

[B34] Cui C. B. Kakeya H. Osada H. (1996). Novel mammalian cell cycle inhibitors, spirotryprostatins A and B, produced by *Aspergillus fumigatus*, which inhibit mammalian cell cycle at G2/M phase. *Tetrahedron* 52 12651–12666. 10.1016/0040-4020(96)00737-5

[B35] Dashti Y. Grkovic T. Abdelmohsen U. R. Hentschel U. Quinn R. J. (2014). Production of induced secondary metabolites by a co-culture of sponge-associated actinomycetes, *Actinokineospora* sp. EG49 and *Nocardiopsis* sp. RV163. *Mar. Drugs* 12 3046–3059. 10.3390/md12053046 24857962 PMC4052330

[B36] Degenkolb T. Heinze S. Schlegel B. Strobel G. Grafe U. (2002). Formation of new lipoaminopeptides, acremostatins A, B, and C, by co-cultivation of *Acremonium* sp. tbp-5 and *Mycogone rosea* DSM 12973. *Biosci. Biotechnol. Biochem.* 66 883–886. 10.1271/bbb.66.883 12036069

[B37] Deng Z. L. Du C. X. Li X. Hu B. Kuang Z. K. Wang R. (2013). Exploring the biologically relevant chemical space for drug discovery. *J. Chem. Inf. Model.* 53 2820–2828. 10.1021/ci400432a 24125686

[B38] Dinarvand M. Rezaee M. Masomian M. Jazayeri S. D. Zareian M. Abbasi S. (2013). Effect of C/N ratio and media optimization through response surface methodology on simultaneous productions of intra- and extracellular inulinase and invertase from *Aspergillus niger* ATCC 20611. *Biomed Res. Int.* 2013:508968. 10.1155/2013/508968 24151605 PMC3787555

[B39] Ding G. Liu S. C. Guo L. D. Zhou Y. G. Che Y. S. (2008). Antifungal metabolites from the plant endophytic fungus *Pestalotiopsis foedan*. *J. Nat. Prod.* 71 615–618. 10.1021/np070590f 18288805

[B40] Ding G. Qi Y. Liu S. Guo L. Chen X. (2012). Photipyrones A and B, new pyrone derivatives from the plant endophytic fungus *Pestalotiopsis photiniae*. *J. Antibiot.* 65 271–273. 10.1038/ja.2012.14 22415458

[B41] Ding G. Zheng Z. H. Liu S. C. Zhang H. Guo L. D. Che Y. S. (2009). Photinides A-F, cytotoxic benzofuranone-derived gamma-lactones from the plant endophytic fungus *Pestalotiopsis photiniae*. *J. Nat. Prod.* 72 942–945. 10.1021/np900084d 19371070

[B42] Ding H. Wang J. N. Zhang D. S. Ma Z. J. (2017). Derivatives of holomycin and cyclopropaneacetic acid from *Streptomyces* sp. DT-A37. *Chem. Biodivers.* 14:e1700140. 10.1002/cbdv.201700140 28627048

[B43] Dong Y. S. Lin J. Lu X. H. Zheng Z. H. Ren X. Zhang H. (2009). Cathepsin B inhibitory tetraene lactones from the fungus *Talaromyces wortmannii*. *Helv. Chim. Acta* 92 567–574. 10.1002/hlca.200800333

[B44] Dong Y. S. Yang J. S. Zhang H. Lin J. Ren X. Liu M. (2006). Wortmannilactones A-D, 22-membered triene macrolides from *Talaromyces wortmannii*. *J. Nat. Prod.* 69 128–130. 10.1021/np0502894 16441083

[B45] Du L. Feng T. Zhao B. Y. Li D. H. Cai S. X. Zhu T. J. (2010). Alkaloids from a deep ocean sediment-derived fungus *Penicillium* sp and their antitumor activities. *J. Antibiot.* 63 165–170. 10.1038/ja.2010.11 20186171

[B46] Du L. King J. B. Cichewicz R. H. (2014). Chlorinated polyketide obtained from a *Daldinia* sp. treated with the epigenetic modifier suberoylanilide hydroxamic acid. *J. Nat. Prod.* 77 2454–2458. 10.1021/np500522z 25338315 PMC4251535

[B47] Du L. Li D. H. Zhu T. J. Cai S. X. Wang F. P. Xiao X. (2009). New alkaloids and diterpenes from a deep ocean sediment derived fungus *Penicillium* sp. *Tetrahedron* 65 1033–1039. 10.1016/j.tet.2008.11.078

[B48] Ebrahim W. El-Neketi M. Lewald L. I. Orfali R. S. Lin W. H. Rehberg N. (2016). Metabolites from the fungal endophyte *Aspergillus austroafricanus* in axenic culture and in fungal-bacterial mixed cultures. *J. Nat. Prod.* 79 914–922. 10.1021/acs.jnatprod.5b00975 27070198

[B49] Elnaggar M. S. Ebada S. S. Ashour M. L. Ebrahim W. Müller W. E. G. Mándi A. (2016). Xanthones and sesquiterpene derivatives from a marine-derived fungus *Scopulariopsis* sp. *Tetrahedron* 72 2411–2419. 10.1016/j.tet.2016.03.073

[B50] Elnaggar M. S. Ebada S. S. Ashour M. L. Ebrahim W. Singab A. Lin W. (2017). Two new triterpenoids and a new naphthoquinone derivative isolated from a hard coral-derived fungus *Scopulariopsis* sp. *Fitoterapia* 116 126–130. 10.1016/j.fitote.2016.12.003 27932272

[B51] Elsayed S. S. Trusch F. Deng H. Raab A. Prokes I. Busarakam K. (2015). Chaxapeptin, a lasso peptide from extremotolerant *Streptomyces leeuwenhoekii* strain C58 from the hyperarid Atacama desert. *J. Org. Chem.* 80 10252–10260. 10.1021/acs.joc.5b01878 26402731

[B52] Ezaki M. Iwami M. Yamashita M. Komori T. Umehara K. Imanaka H. (1992). Biphenomycin A production by a mixed culture. *Appl. Environ. Microbiol.* 58 3879–3882. 1476432 10.1128/aem.58.12.3879-3882.1992 PMC183198

[B53] Ezaki M. Shigematsu N. Yamashita M. Komori T. Umehara K. Imanaka H. (1993). Biphenomycin C, a precursor of biphenomycin A in mixed culture. *J. Antibiot.* 46 135–140. 10.7164/antibiotics.46.135 8436546

[B54] Fang S. M. Wu C. J. Li C. W. Cui C. B. (2014). A practical strategy to discover new antitumor compounds by activating silent metabolite production in fungi by diethyl sulphate mutagenesis. *Mar. Drugs* 12 1788–1814. 10.3390/md12041788 24681631 PMC4012455

[B55] Feller G. Narinx E. Arpigny J. L. Zekhnini Z. Swings J. Gerday C. (1994). Temperature dependence of growth, enzyme secretion and activity of psychrophilic antarctic bacteria. *Appl. Microbiol. Biotechnol.* 41 477–479. 10.1007/BF00939039

[B56] Fuchser J. Thiericke R. Zeeck A. (1995). Biosynthesis of aspinonene, a branched pentaketide produced by *Aspergillus ochraceus*, Related to aspyrone. *J. Chem. Soc. Perkin Trans.* 1 1663–1666. 10.1039/p19950001663

[B57] Fukuda H. Sano N. Muto S. Horikoshi M. (2006). Simple histone acetylation plays a complex role in the regulation of gene expression. *Brief. Funct. Genomic. Proteomic.* 5 190–208. 10.1093/bfgp/ell032 16980317

[B58] Gao H. Zhou L. Cai S. Zhang G. Zhu T. Gu Q. (2013). Diorcinols B-E, new prenylated diphenyl ethers from the marine-derived fungus *Aspergillus versicolor* ZLN-60. *J. Antibiot.* 66 539–542. 10.1038/ja.2013.40 23677033

[B59] Gao S. S. Li X. M. Zhang Y. Li C. S. Cui C. M. Wang B. G. (2011). Comazaphilones A-F, azaphilone derivatives from the marine sediment-derived fungus *Penicillium commune* QSD-17. *J. Nat. Prod.* 74 256–261. 10.1021/np100788h 21226488

[B60] Gao S. S. Shang Z. Li X. M. Li C. S. Cui C. M. Wang B. G. (2012). Secondary metabolites produced by solid fermentation of the marine-derived fungus *Penicillium commune* QSD-17. *Biosci. Biotechnol. Biochem.* 76 358–360. 10.1271/bbb.110332 22313755

[B61] Gibson A. M. Bratchell N. Roberts T. A. (1988). Predicting microbial growth: growth responses of salmonellae in a laboratory medium as affected by pH, sodium chloride and storage temperature. *Int. J. Food Microbiol.* 6 155–178. 10.1016/0168-1605(88)90051-7 3275296

[B62] Grond S. Papastavrou I. Zeeck A. (2000). Studies of precursor-directed biosynthesis with streptomyces, 3 - structural diversity of 1-o-acyl alpha-l-rhamnopyranosides by precursor-directed biosynthesis with *Streptomyces griseoviridis*. *Eur. J. Org. Chem.* 10 1875–1881. 10.1002/(SICI)1099-0690(200005)2000:10<1875::AID-EJOC1875>3.0.CO;2-G

[B63] Grond S. Papastavrou I. Zeeck A. (2002). Studies of precursor-directed biosynthesis with streptomyces, part 4. Novel alpha-L-rhamnopyranosides from a single strain of Streptomyces by supplement-induced biosynthetic steps. *Eur. J. Org. Chem.* 19 3237–3242. 10.1002/1099-0690(200210)2002:19<3237::AID-EJOC3237>3.0.CO;2-T

[B64] Gulder T. A. M. Hong H. Correa J. Egereva E. Wiese J. Imhoff J. F. (2012). Isolation, structure elucidation and total synthesis of lajollamide a from the marine fungus *Asteromyces cruciatus*. *Mar. Drugs* 10 2912–2935. 10.3390/md10122912 23342379 PMC3528133

[B65] Gunatilaka A. A. L. (2006). Natural products from plant-associated microorganisms: distribution, structural diversity, bioactivity, and implications of their occurrence. *J. Nat. Prod.* 69 509–526. 10.1021/np058128n 16562864 PMC3362121

[B66] Guo W. Peng J. Zhu T. Gu Q. Keyzers R. A. Li D. (2013). Sorbicillamines A-E, nitrogen-containing sorbicillinoids from the deep-sea-derived fungus *Penicillium* sp. F23-2. *J. Nat. Prod.* 76 2106–2112. 10.1021/np4006647 24215398

[B67] Hemphill C. F. P. Sureechatchaiyan P. Kassack M. U. Orfali R. S. Lin W. Daletos G. (2017). OSMAC approach leads to new fusarielin metabolites from *Fusarium tricinctum*. *J. Antibiot.* 70 726–732. 10.1038/ja.2017.21 28270687

[B68] Henrikson J. C. Hoover A. R. Joyner P. M. Cichewicz R. H. (2009). A chemical epigenetics approach for engineering the in situ biosynthesis of a cryptic natural product from *Aspergillus niger*. *Org. Biomol. Chem.* 7 435–438. 10.1039/b819208a 19156306

[B69] Hewage R. T. Aree T. Mahidol C. Ruchirawat S. Kittakoop P. (2014). One strain-many compounds (OSMAC) method for production of polyketides, azaphilones, and an isochromanone using the endophytic fungus *Dothideomycete* sp. *Phytochemstry* 108 87–94. 10.1016/j.phytochem.2014.09.013 25310919

[B70] Hoshino S. Okada M. Awakawa T. Asamizu S. Onaka H. Abe I. (2017). Mycolic acid containing bacterium stimulates tandem cyclization of polyene macrolactam in a lake sediment derived rare *Actinomycete*. *Org. Lett.* 19 4992–4995. 10.1021/acs.orglett.7b02508 28880091

[B71] Hoshino S. Okada M. Wakimoto T. Zhang H. Hayashi F. Onaka H. (2015). Niizalactams A-C, multicyclic macrolactams isolated from combined culture of *Streptomyces* with mycolic acid-containing bacterium. *J. Nat. Prod.* 78 3011–3017. 10.1021/acs.jnatprod.5b00804 26624939

[B72] Hozzein W. N. Li W. J. Ali M. I. Hammouda O. Mousa A. S. Xu L. H. (2004). *Nocardiopsis alkaliphila* sp. nov., a novel alkaliphilic actinomycete isolated from desert soil in Egypt. *Int. J. Syst. Evol. Microbiol.* 54 247–252. 10.1099/ijs.0.02832-0 14742488

[B73] Hug J. J. Bader C. D. Remškar M. Cirnski K. Müller R. (2018). Concepts and methods to access novel antibiotics from actinomycetes. *Antibiotics* 7:E44. 10.3390/antibiotics7020044 29789481 PMC6022970

[B74] Hussain A. Rather M. A. Dar M. S. Aga M. A. Ahmad N. Manzoor A. (2017). Novel bioactive molecules from *Lentzea violacea* strain AS 08 using one strain-many compounds (OSMAC) approach. *Bioorg. Med. Chem. Lett.* 27 2579–2582. 10.1016/j.bmcl.2017.03.075 28400238

[B75] Jiang W. Zhong Y. Q. Shen L. Wu X. D. Ye Y. Chen C. T. A. (2014). Stress-driven discovery of natural products from extreme marine environment-Kueishantao hydrothermal vent, a case study of metal switch valve. *Curr. Org. Chem.* 18 925–934. 10.2174/138527281807140515155705

[B76] Kakeya H. Morishita M. Onozawa C. Usami R. Horikoshi K. Kimura K. (1997). RKS-1778, a new mammalian cell-cycle inhibitor and a key intermediate of the 11 cytochalasin group. *J. Nat. Prod.* 60 669–672. 10.1021/np970151o 9249969

[B77] Kamauchi H. Kinoshita K. Sugita T. Koyama K. (2016). Conditional changes enhanced production of bioactive metabolites of marine derived fungus *Eurotium rubrum*. *Bioorg. Med. Chem. Lett.* 26 4911–4914. 10.1016/j.bmcl.2016.09.017 27641468

[B78] Karakoç S. B. Aksöz N. (2004). Optimization of carbon-nitrogen ratio for production of gibberellic acid by *Pseudomonas* sp. *Pol. J. Microbiol.* 53 117–120. 15478357

[B79] Kozone I. Ueda J. Takagi M. Shin-ya K. (2009). JBIR-52, a new antimycin-like compound from *Streptomyces* sp. ML55. *J. Antibiot.* 62 593–595. 10.1038/ja.2009.79 19662084

[B80] Kurosawa K. Ghiviriga I. Sambandan T. G. Lessard P. A. Barbara J. E. Rha C. (2008). Rhodostreptomycins, antibiotics biosynthesized following horizontal gene transfer from *Streptomyces padanus* to *Rhodococcus fascians*. *J. Am. Chem. Soc.* 130 1126–1127. 10.1021/ja077821p 18179219

[B81] Li C. S. An C. Y. Li X. M. Gao S. S. Cui C. M. Sun H. F. (2011). Triazole and dihydroimidazole alkaloids from the marine sediment-derived fungus *Penicillium paneum* SD-44. *J. Nat. Prod.* 74 1331–1334. 10.1021/np200037z 21495659

[B82] Li C. S. Li X. M. Gao S. S. Lu Y. H. Wang B. G. (2013). Cytotoxic anthranilic acid derivatives from deep sea sediment-derived fungus *Penicillium paneum* SD-44. *Mar. Drugs* 11 3068–3076. 10.3390/md11083068 23966037 PMC3766882

[B83] Li X. Zvanych R. Torchia J. Magarvey N. A. (2013). Structures and biosynthesis of 12-membered macrocyclic depsipeptides from *Streptomyces* sp. ML55. *Bioorg. Med. Chem. Lett.* 23 4150–4153. 10.1016/j.bmcl.2013.05.042 23756369

[B84] Li G. Kusari S. Golz C. Laatsch H. Strohmann C. Spiteller M. (2017). Epigenetic modulation of endophytic *Eupenicillium* sp. LG41 by a histone deacetylase inhibitor for production of decalin-containing compounds. *J. Nat. Prod.* 80 983–988. 10.1021/acs.jnatprod.6b00997 28333449

[B85] Li X. Xia Z. Tang J. Wu J. Tong J. Li M. (2017). Identification and biological evaluation of secondary metabolites from marine derived fungi *Aspergillus* sp. SCSIOW3, cultivated in the presence of epigenetic modifying agents. *Molecules* 22:E1302. 10.3390/molecules22081302 28777319 PMC6152046

[B86] Li Y. L. Wang J. F. He W. J. Lin X. P. Zhou X. J. Liu Y. H. (2017). One strain-many compounds method for production of polyketide metabolites using the sponge-derived fungus *Arthrinium arundinis* ZSDS1-F3. *Chem. Nat. Compd.* 53 373–374. 10.1007/s10600-017-1994-3

[B87] Li J. Lu C. Shen Y. (2008). Novel polyketides isolated from *Streptomyces* sp. *Helv. Chim. Acta* 91 741–745. 10.1002/hlca.200890075

[B88] Li J. Lu C. Shen Y. (2010). Macrolides of the bafilomycin family produced by *Streptomyces* sp. CS. *J. Antibiot.* 63 595–599. 10.1038/ja.2010.95 20823894

[B89] Lin Z. J. Zhu T. J. Chen L. Gu Q. Q. (2010). Three new aspochalasin derivatives from the marine-derived fungus *Spicaria elegans*. *Chin. Chem. Lett.* 21 824–826. 10.1016/j.cclet.2010.02.019

[B90] Lin Z. J. Zhu T. J. Wei H. J. Zhang G. J. Wang H. Gu Q. Q. (2009a). Spicochalasin A and new aspochalasins from the marine-derived fungus *Spicaria elegans*. *Eur. J. Org. Chem.* 18 3045–3051. 10.1002/ejoc.200801085

[B91] Lin Z. J. Zhu T. J. Zhang G. J. Wei H. J. Gu Q. Q. (2009b). Deoxy-cytochalasins from a marine-derived fungus *Spicaria elegans*. *Can. J. Chem.* 87 486–489. 10.1139/v09-006

[B92] Liu C. X. Liu S. H. Zhao J. W. Zhang J. Wang X. J. Li J. S. (2016). A new spectinabilin derivative with cytotoxic activity from ant-derived *Streptomyces* sp. 1H-GS5. *J. Asian Nat. Prod. Res.* 19 924–929. 10.1080/10286020.2016.1254200 27838921

[B93] Liu M. Liu N. Shang F. Huang Y. (2016). Activation and identification of NC-1: a cryptic cyclodepsipeptide from red soil-derived *Streptomyces* sp. FXJ1.172. *Eur. J. Org. Chem.* 2016 3943–3948. 10.1002/ejoc.201600297

[B94] Liu W. C. Yang F. Zhang R. Shi X. Lu X. H. Luan Y. S. (2016). Production of polyketides with anthelmintic activity by the fungus *Talaromyces wortmannii* using one strain-many compounds (OSMAC) method. *Phytochem. Lett.* 18 157–161. 10.1016/j.phytol.2016.10.006

[B95] Liu L. Bruhn T. Guo L. Gotz D. C. Brun R. Stich A. (2011). Chloropupukeanolides C-E: cytotoxic pupukeanane chlorides with a spiroketal skeleton from *Pestalotiopsis fici*. *Chemistry* 17 2604–2613. 10.1002/chem.201003129 21305627

[B96] Liu L. Li Y. Liu S. C. Zheng Z. H. Chen X. L. Zhang H. (2009a). Chloropestolide A, an antitumor metabolite with an unprecedented spiroketal skeleton from *Pestalotiopsis fici*. *Org. Lett.* 11 2836–2839. 10.1021/ol901039m 19496604

[B97] Liu L. Liu S. C. Niu S. B. Guo L. D. Chen X. L. Che Y. S. (2009b). Isoprenylated chromone derivatives from the plant endophytic fungus *Pestalotiopsis fici*. *J. Nat. Prod.* 72 1482–1486. 10.1021/np900308s 19618920

[B98] Liu L. Liu S. C. Jiang L. H. Chen X. L. Guo L. D. Che Y. S. (2008a). Chloropupukeananin, the first chlorinated pupukeanane derivative, and its precursors from *Pestalotiopsis fici*. *Org. Lett.* 10 1397–1400. 10.1021/ol800136t 18314997

[B99] Liu L. Tian R. Liu S. Chen X. Guo L. Che Y. (2008b). Pestaloficiols A-E, bioactive cyclopropane derivatives from the plant endophytic fungus *Pestalotiopsis fici*. *Bioorg. Med. Chem.* 16 6021–6026. 10.1016/j.bmc.2008.04.052 18468908

[B100] Liu R. Lin Z. J. Zhu T. J. Fang Y. C. Gu Q. Q. Zhu W. M. (2008c). Novel open-chain cytochalasins from the marine-derived fungus *Spicaria elegans*. *J. Nat. Prod.* 71 1127–1132. 10.1021/np070539b 18507474

[B101] Liu L. Niu S. Lu X. Chen X. Zhang H. Guo L. (2010). Unique metabolites of *Pestalotiopsis fici* suggest a biosynthetic hypothesis involving a Diels-Alder reaction and then mechanistic diversification. *Chem. Commun.* 46 460–462. 10.1039/b918330b 20066325

[B102] Liu R. Gu Q. Q. Zhu W. M. Cui C. B. Fan G. T. (2005). Trichodermamide A and aspergillazine A, two cytotoxic modified dipeptides from a marine-derived fungus *Spicaria elegans*. *Arch. Pharm. Res.* 28 1042–1046. 10.1007/bf02977399 16212235

[B103] Liu R. Gu Q. Q. Zhu W. M. Cui C. B. Fan G. T. Fang Y. C. (2006). 10-phenyl- 12-cytochalasins Z(7), Z(8), and Z(9) from the marine-derived fungus *Spicaria elegans*. *J. Nat. Prod.* 69 871–875. 10.1021/np050201m 16792402

[B104] Liu S. Dai H. F. Heering C. Janiak C. Lin W. H. Liu Z. (2017). Inducing new secondary metabolites through co-cultivation of the fungus *Pestalotiopsis* sp with the bacterium *Bacillus subtilis*. *Tetrahedron Lett.* 58 257–261. 10.1016/j.tetlet.2016.12.026

[B105] Liu S. Xu M. Zhang H. Qi H. Zhang J. Liu C. (2015). New cytotoxic spectinabilin derivative from ant-associated *Streptomyces* sp. 1H-GS5. *J. Antibiot.* 69 128–131. 10.1038/ja.2015.99 26374562

[B106] Liu Y. Li X. M. Meng L. H. Jiang W. L. Xu G. M. Huang C. G. (2015). Bisthiodiketopiperazines and acorane sesquiterpenes produced by the marine-derived fungus *Penicillium adametzioides* AS-53 n different culture media. *J. Nat. Prod.* 78 1294–1299. 10.1021/acs.jnatprod.5b00102 26039736

[B107] Lu C. Shen Y. (2003). A new macrolide antibiotic with antitumor activity produced by *Streptomyces* sp. CS, a commensal microbe of *Maytenus hookeri*. *J. Antibiot.* 56 415–418. 10.7164/antibiotics.56.415 12817815

[B108] Lu C. Shen Y. (2004). Two new macrolides produced by *Streptomyces* sp. CS. *J. Antibiot.* 57 597–600. 10.7164/antibiotics.57.597 15580961

[B109] Luan Y. P. Wei H. J. Zhang Z. P. Che Q. Liu Y. K. Zhu T. J. (2014). Eleganketal A, a highly oxygenated dibenzospiroketal from the marine-derived fungus *Spicaria elegans* KLA03. *J. Nat. Prod.* 77 1718–1723. 10.1021/np500458a 24967847

[B110] Ma L. Xing D. Wang H. Wang X. Xue D. (2009). Effect of culture conditions on cell growth and lipid accumulation of oleaginous microorganism. *Chin. J. Biotechnol.* 25 55–59. 19441227

[B111] Marmann A. Aly A. H. Lin W. H. Wang B. G. Proksch P. (2014). Co-cultivation a powerful emerging tool for enhancing the chemical diversity of microorganisms. *Mar. Drugs* 12 1043–1065. 10.3390/md12021043 24549204 PMC3944530

[B112] Meng L. H. Li X. M. Liu Y. Xu G. M. Wang B. G. (2017). Antimicrobial alkaloids produced by the mangrove endophyte *Penicillium brocae* MA-231 using the OSMAC approach. *RSC Adv.* 7 55026–55033. 10.1039/c7ra12081h

[B113] Meng L. H. Li X. M. Lv C. T. Huang C. G. Wang B. G. (2014). Brocazines A-F, cytotoxic bisthiodiketopiperazine derivatives from *Penicillium brocae* MA-231, an endophytic fungus derived from the marine mangrove plant *Avicennia marina*. *J. Nat. Prod.* 77 1921–1927. 10.1021/np500382k 25105722

[B114] Meng L. H. Liu Y. Li X. M. Xu G. M. Ji N. Y. Wang B. G. (2015a). Citrifelins A and B, citrinin adducts with a tetracyclic framework from cocultures of marine-derived isolates of *Penicillium citrinum* and *Beauveria felina*. *J. Nat. Prod.* 78 2301–2305. 10.1021/acs.jnatprod.5b00450 26295595

[B115] Meng L. H. Zhang P. Li X. M. Wang B. G. (2015b). Penicibrocazines A-E, five new sulfide diketopiperazines from the marine-derived endophytic fungus *Penicillium brocae*. *Mar. Drugs* 13 276–287. 10.3390/md13010276 25574740 PMC4306937

[B116] Miao F. P. Liang X. R. Liu X. H. Ji N. Y. (2014). Aspewentins A-C, norditerpenes from a cryptic pathway in an algicolous strain of *Aspergillus wentii*. *J. Nat. Prod.* 77 429–432. 10.1021/np401047w 24499164

[B117] Moore J. M. Bradshaw E. Seipke R. F. Hutchings M. I. McArthur M. (2012). Use and discovery of chemical elicitors that stimulate biosynthetic gene clusters in *Streptomyces* bacteria. *Methods Enzymol.* 517 367–385. 10.1016/B978-0-12-404634-4.00018-8 23084948

[B118] Ochi K. Okamoto S. Tozawa Y. Inaoka T. Hosaka T. Xu J. (2004). Ribosome engineering and secondary metabolite production. *Adv. Appl. Microbiol.* 56 155–184. 10.1016/S0065-2164(04)56005-7 15566979

[B119] Oh D. C. Jensen P. R. Kauffman C. A. Fenical W. (2005). Libertellenones A-D: induction of cytotoxic diterpenoid biosynthesis by marine microbial competition. *Bioorg. Med. Chem.* 13 5267–5273. 10.1016/j.bmc.2005.05.068 15993608

[B120] Oh D. C. Kauffman C. A. Jensen P. R. Fenical W. (2007). Induced production of emericellamides A and B from the marine-derived fungus *Emericella* sp. in competing co-culture. *J. Nat. Prod.* 70 515–520. 10.1021/np060381f 17323993

[B121] Oikawa H. Murakami Y. Ichihara A. (1992). Useful approach to find the plausible biosynthetic precursors of secondary metabolites using P-450 inhibitors-postulated intermediates of chaetoglobosin-A1. *J. Chem. Soc. Perkin Trans.* 1 2949–2953. 10.1039/p19920002949

[B122] Ola A. R. Thomy D. Lai D. Brotz-Oesterhelt H. Proksch P. (2013). Inducing secondary metabolite production by the endophytic fungus *Fusarium tricinctum* through coculture with *Bacillus subtilis*. *J. Nat. Prod.* 76 2094–2099. 10.1021/np400589h 24175613

[B123] Onaka H. (2017). Novel antibiotic screening methods to awaken silent or cryptic secondary metabolic pathways in actinomycetes. *J. Antibiot.* 70 865–870. 10.1038/ja.2017.51 28442735

[B124] Onaka H. Mori Y. Igarashi Y. Furumai T. (2011). Mycolic acid-containing bacteria induce natural-product biosynthesis in *Streptomyces* species. *Appl. Environ. Microbiol.* 77 400–406. 10.1128/AEM.01337-10 21097597 PMC3020563

[B125] Overy D. P. Zidorn C. Petersen B. O. Duus J. Ø. Dalsgaard P. W. Larsen T. O. (2005). Medium dependant production of corymbiferone a novel product from *Penicillium hordei* cultured on plant tissue agar. *Tetrahedron Lett.* 46 3225–3228. 10.1016/j.tetlet.2005.03.043

[B126] Paranagama P. A. Wijeratne E. M. K. Gunatilaka A. A. L. (2007). Uncovering biosynthetic potential of plant-associated fungi: effect of culture conditions on metabolite production by *Paraphaeosphaeria quadriseptata* and *Chaetomium chiversii*. *J. Nat. Prod.* 70 1939–1945. 10.1021/np070504b 18052326

[B127] Park H. B. Park J. S. Lee S. I. Shin B. Oh D. C. Kwon H. C. (2017). Gordonic acid, a polyketide glycoside derived from bacterial coculture of *Streptomyces* and *Gordonia* Species. *J. Nat. Prod.* 80 2542–2546. 10.1021/acs.jnatprod.7b00293 28845982

[B128] Peng J. X. Gao H. Q. Zhang X. M. Wang S. Wu C. M. Gu Q. Q. (2014). Psychrophilins E-H and versicotide C, cyclic peptides from the marine-derived fungus *Aspergillus versicolor* ZLN-60. *J. Nat. Prod.* 77 2218–2223. 10.1021/np500469b 25246036

[B129] Peng X. P. Wang Y. Liu P. P. Hong K. Chen H. Yin X. (2011). Aromatic compounds from the halotolerant fungal strain of *Wallemia sebi* PXP-89 in a hypersaline medium. *Arch. J. Pharm. Res.* 34 907–912. 10.1007/s12272-011-0607-0 21725811

[B130] Pérez J. Braña A. F. Shimkets L. J. Sevillano L. Santamaría R. I. (2011). *Myxococcus xanthus* induces actinorhodin overproduction and aerial mycelium formation by *Streptomyces coelicolor*. *Microb. Biotechnol.* 4 175–183. 10.1111/j.1751-7915.2010.00208.x 21342463 PMC3818858

[B131] Poolman B. Glaasker E. (1998). Regulation of compatible solute accumulation in bacteria. *Mol. Microbiol.* 29 397–407. 10.1046/j.1365-2958.1998.00875.x 9720860

[B132] Puder C. Loya S. Hizi A. Zeeck A. (2001). New co-metabolites of the streptazolin pathway. *J. Nat. Prod.* 64 42–45. 10.1021/np000377i 11170664

[B133] Ramm S. Krawczyk B. Mühlenweg A. Poch A. Mösker E. Süssmuth R. D. (2017). A self-sacrificing n-methyltransferase is the precursor of the fungal natural product omphalotin. *Angew. Chem. Int. Ed.* 56 9994–9997. 10.1002/anie.201703488 28715095

[B134] Rateb M. E. Hallyburton I. Houssen W. E. Bull A. T. Goodfellow M. Santhanam R. (2013). Induction of diverse secondary metabolites in *Aspergillus fumigatus* by microbial co-culture. *RSC Adv.* 3 14444–14450. 10.1039/c3ra42378f

[B135] Rateb M. E. Houssen W. E. Arnold M. Abdelrahman M. H. Deng H. Harrison W. T. (2011a). Chaxamycins A-D, bioactive ansamycins from a hyper-arid desert *Streptomyces* sp. *J. Nat. Prod.* 74 1491–1499. 10.1021/np200320u 21553813

[B136] Rateb M. E. Houssen W. E. Harrison W. T. A. Deng H. Okoro C. K. Asenjo J. A. (2011b). Diverse metabolic profiles of a *Streptomyces* strain isolated from a hyper-arid environment. *J. Nat. Prod.* 74 1965–1971. 10.1021/np200470u 21879726

[B137] Reid K. A. Hamilton J. T. G. Bowden R. D. O’Hagan D. Dasaradhi L. Amin M. R. (1995). Biosynthesis of fluorinated secondary metabolites by *Streptomyces cattleya*. *Microbiology* 141 1385–1393. 10.1099/13500872-141-6-1385 7670640

[B138] Ruiz B. Chavez A. Forero A. Garca-Huante Y. Romero A. Sanchez M. (2009). Production of microbial secondary metabolites: regulation by the carbon source. *Crit. Rev. Miocrbiol.* 36 146–167. 10.3109/10408410903489576 20210692

[B139] Sato S. (1990). Microbial production and control of cellular growth under high dissolved oxygen concentration. *J. Ferment. Bioeng.* 70:293 10.1016/0922-338x(90)90076-9

[B140] Schäberle T. F. Orland A. König G. M. (2014). Enhanced production of undecylprodigiosin in *Streptomyces coelicolor* by co-cultivation with the corallopyronin A-producing myxobacterium, *Corallococcus coralloides*. *Biotechnol. Lett.* 36 641–648. 10.1007/s10529-013-1406-0 24249103

[B141] Scherlach K. Hertweck C. (2009). Triggering cryptic natural product biosynthesis in microorganisms. *Org. Biomol. Chem.* 7 1753–1760. 10.1039/b821578b 19590766

[B142] Schneider P. Misiek M. Hoffmeister D. (2008). In vivo and in vitro production options for fungal secondary metabolites. *Mol. Pharm.* 5 234–242. 10.1021/mp7001544 18330989

[B143] Schroeckh V. Scherlach K. Nutzmann H. W. Shelest E. Schmidt-Heck W. Schuemann J. (2009). Intimate bacterial-fungal interaction triggers biosynthesis of archetypal polyketides in *Aspergillus nidulans*. *Proc. Natl. Acad. Sci.* 106 14558–14563. 10.1073/pnas.0901870106 19666480 PMC2732885

[B144] Senadeera S. P. Wiyakrutta S. Mahidol C. Ruchirawat S. Kittakoop P. (2012). A novel tricyclic polyketide and its biosynthetic precursor azaphilone derivatives from the endophytic fungus *Dothideomycete* sp. *Org. Biomol. Chem.* 10 7220–7226. 10.1039/c2ob25959a 22847560

[B145] Seyedsayamdost M. R. (2014). High-throughput platform for the discovery of elicitors of silent bacterial gene clusters. *Proc. Natl. Acad Sci. U.S.A.* 111 7266–7271. 10.1073/pnas.1400019111 24808135 PMC4034229

[B146] Shang Z. Salim A. A. Capon R. J. (2017). Chaunopyran a: co-cultivation of marine mollusk-derived fungi activates a rare class of 2-alkenyl-tetrahydropyran. *J. Nat. Prod.* 80 1167–1172. 10.1021/acs.jnatprod.7b00144 28383912

[B147] Shin C. S. Kim H. J. Kim M. J. Ju J. Y. (1998). Morphological change and enhanced pigment production of *Monascus* when cocultured with *Saccharomyces cerevisiae* or *Aspergillus oryzae*. *Biotechnol. Bioeng.* 59 576–581. 10.1002/(SICI)1097-0290(19980905)59:5<576::AID-BIT7>3.0.CO;2-7 10099374

[B148] Singh V. Haque S. Niwas R. Srivastava A. Pasupuleti M. Tripathi C. K. M. (2017). Strategies for fermentation medium optimization: an indepth review. *Front. Microbiol.* 7:2087 10.3389/fmicb.2016.02087 PMC5216682 28111566

[B149] Siridechakorn I. Yue Z. Mittraphab Y. Lei X. Pudhom K. (2017). Identification of spirobisnaphthalene derivatives with anti-tumor activities from the endophytic fungus *Rhytidhysteron rufulum* AS21B. *Bioorg. Med. Chem.* 25 2878–2882. 10.1016/j.bmc.2017.02.054 28274675

[B150] Suh J. H. Shin C. S. (2000a). Analysis of the morphologic changes of *Monascus* sp J101 cells cocultured with *Saccharomyces cerevisiae*. *FEMS Microbiol. Lett.* 193 143–147. 10.1016/s0378-1097(00)00470-5 11094293

[B151] Suh J. H. Shin C. S. (2000b). Physiological analysis on novel coculture of *Monascus* sp J101 with *Saccharomyces cerevisiae*. *FEMS Microbiol. Lett.* 190 241–245. 10.1111/j.1574-6968.2000.tb09293.x 11034286

[B152] Sun J. Awakawa T. Noguchi H. Abe I. (2012). Induced production of mycotoxins in an endophytic fungus from the medicinal plant *Datura stramonium* L. *Bioorg. Med. Chem. Lett.* 22 6397–6400. 10.1016/j.bmcl.2012.08.063 22967766

[B153] Sureram S. Kesornpun C. Mahidol C. Ruchirawat S. Kittakoop P. (2013). Directed biosynthesis through biohalogenation of secondary metabolites of the marine-derived fungus *Aspergillus unguis*. *RSC Adv.* 3 1781–1788. 10.1039/c2ra23021f

[B154] Sureram S. Wiyakrutta S. Ngamrojanavanich N. Mahidol C. Ruchirawat S. Kittakoop P. (2012). Depsidones, aromatase inhibitors and radical scavenging agents from the marine-derived fungus *Aspergillus unguis* CRI282-03. *Planta Med.* 78 582–588. 10.1055/s-0031-1298228 22307935

[B155] Tammen S. A. Friso S. Choi S.-W. (2013). Epigenetics: the link between nature and nurture. *Mol. Aspects Med.* 34 753–764. 10.1016/j.mam.2012.07.018 22906839 PMC3515707

[B156] Tan Y. Wang Z. Marshall K. C. (1998). Modeling pH effects on microbial growth: a statistical thermodynamic approach. *Biotechnol. Bioeng.* 59 724–731. 10.1002/(SICI)1097-0290(19980920)59:6<724::AID-BIT9>3.0.CO;2-H 10099393

[B157] Tang J. W. Wang W. G. Li A. Yan B. C. Chen R. Li X. N. (2017). Polyketides from the endophytic fungus *Phomopsis* sp sh917 by using the one strain/many compounds strategy. *Tetrahedron* 73 3577–3584. 10.1016/j.tet.2017.02.019

[B158] Teles A. P. Takahashi J. A. (2013). Paecilomide, a new acetylcholinesterase inhibitor from *Paecilomyces lilacinus*. *Microbiol. Res.* 168 204–210. 10.1016/j.micres.2012.11.007 23219197

[B159] Thorneley R. N. F. (1990). *Metal Ions and Bacteria* Vol. 8. Amsterdam: Elsevier Ltd 298–299. 10.1016/0167-7799(90)90204-B

[B160] Traxler M. F. Watrous J. D. Alexandrov T. Dorrestein P. C. Kolter R. (2013). Interspecies interactions stimulate diversification of the *Streptomyces coelicolor* secreted metabolome. *mBio* 4:e00459-13. 10.1128/mBio.00459-13 23963177 PMC3747584

[B161] Uchida I. Uchida I. Shigematsu N. Shigematsu N. Ezaki M. Ezaki M. (1985). Biphenomycins A and B, novel peptide antibiotics II. Structural elucidation of biphenomycins A and B. *J. Antibiot.* 38 1462–1468. 10.7164/antibiotics.38.1462 3841123

[B162] Uchoa P. K. S. Pimenta A. T. A. Braz-Filho R. de Oliveira M. Saraiva N. N. Rodrigues B. S. F. (2017). New cytotoxic furan from the marine sediment-derived fungi *Aspergillus niger*. *Nat. Prod. Res.* 31 2599–2603. 10.1080/14786419.2017.1283499 28135874

[B163] Ueda J. Nagai A. Izumikawa M. Chijiwa S. Takagi M. Shin-ya K. (2008). A novel antimycin-like compound, JBIR-06, from *Streptomyces* sp. ML55. *J. Antibiot.* 61 241–244. 10.1038/ja.2008.35 18503204

[B164] Ueda J. Togashi T. Matukura S. Nagai A. Nakashima T. Komaki H. (2007). A novel nuclear export inhibitor JBIR-02, a new piericidin discovered from *Streptomyces* sp. ML55. *J. Antibiot.* 60 459–462. 10.1038/ja.2007.59 17721005

[B165] Valente A. Ferreira A. G. Daolio C. Rodrigues E. Boffo E. F. Souza A. Q. L. (2013). Production of 5-hydroxy-7-methoxy-4-methylphthalide in a culture of *Penicillium crustosum*. *An. Acad. Bras. Cienc.* 85 487–496. 10.1590/s0001-37652013005000024 23780307

[B166] Vervoort H. C. Draskovic M. Crews P. (2011). Histone deacetylase inhibitors as a tool to up-regulate new fungal biosynthetic products: isolation of EGM-556, a cyclodepsipeptide, from *Microascus* sp. *Org. Lett.* 13 410–413. 10.1021/ol1027199 21174394 PMC3031758

[B167] Wakefield J. Hassan H. M. Jaspars M. Ebel R. Rateb M. E. (2017). Dual induction of new microbial secondary metabolites by fungal bacterial co-cultivation. *Front. Microbiol.* 8:1284. 10.3389/fmicb.2017.01284 28744271 PMC5504103

[B168] Wang B. Park E. M. King J. B. Mattes A. O. Nimmo S. L. Clendinen C. (2015a). Transferring fungi to a deuterium-enriched medium results in assorted, conditional changes in secondary metabolite production. *J. Nat. Prod.* 78 1415–1421. 10.1021/acs.jnatprod.5b00337 26061478 PMC7676450

[B169] Wang F. Z. Wei H. J. Zhu T. J. Li D. H. Lin Z. J. Gu Q. Q. (2011). Three new cytochalasins from the marine-derived fungus *Spicaria elegans* KLA03 by supplementing the cultures with *L*- and *D*-tryptophan. *Chem. Biodivers.* 8 887–894. 10.1002/cbdv.201000133 21560237

[B170] Wang J. Wang Z. Ju Z. R. Wan J. T. Liao S. R. Lin X. P. (2015b). Cytotoxic cytochalasins from marine-derived fungus *Arthrinium arundinis*. *Planta Med.* 81 160–166. 10.1055/s-0034-1383403 25626143

[B171] Wang J. Wei X. Y. Qin X. C. Lin X. P. Zhou X. F. Liao S. R. (2015c). Arthpyrones A-C, pyridone alkaloids from a sponge-derived fungus *Arthrinium arundinis* ZSDS1-F3. *Org. Lett.* 17 656–659. 10.1021/ol503646c 25606827

[B172] Wang J. F. Xu F. Q. Wang Z. Lu X. Wan J. T. Yang B. (2014). A new naphthalene glycoside from the sponge-derived fungus *Arthrinium* sp ZSDS1-F3. *Nat. Prod. Res.* 28 1070–1074. 10.1080/14786419.2014.905935 24735402

[B173] Wang Q. X. Bao L. Yang X. L. Guo H. Ren B. Guo L. D. (2013a). Tricycloalternarenes F-H: three new mixed terpenoids produced by an endolichenic fungus *Ulocladium* sp. using OSMAC method. *Fitoterapia* 85 8–13. 10.1016/j.fitote.2012.12.029 23313270

[B174] Wang Q. X. Bao L. Yang X. L. Guo H. Yang R. N. Ren B. A. (2012). Polyketides with antimicrobial activity from the solid culture of an endolichenic fungus *Ulocladium* sp. *Fitoterapia* 83 209–214. 10.1016/j.fitote.2011.10.013 22061662

[B175] Wang Q. X. Bao L. Yang X. L. Liu D. L. Guo H. Dai H. Q. (2013b). Ophiobolins P-T, five new cytotoxic and antibacterial sesterterpenes from the endolichenic fungus *Ulocladium* sp. *Fitoterapia* 90 220–227. 10.1016/j.fitote.2013.08.002 23954177

[B176] Wang Y. Lu Z. Sun K. Zhu W. (2011). Effects of high salt stress on secondary metabolite production in the marine-derived fungus *Spicaria elegans*. *Mar. Drugs* 9 535–542. 10.3390/md9040535 21731548 PMC3124971

[B177] Wang Z. Fu P. Liu P. P. Wang P. Hou J. B. Li W. J. (2013c). New pyran-2-ones from alkalophilic actinomycete, *Nocardiopsis alkaliphila* sp. Nov. YIM-80379. *Chem. Biodivers.* 10 281–287. 10.1002/cbdv.201200086 23418175

[B178] Wang L. Li M. Tang J. Li X. (2016). Eremophilane sesquiterpenes from a deep marine-derived fungus, *Aspergillus* sp. SCSIOW2, cultivated in the presence of epigenetic modifying agents. *Molelules* 21:473. 10.3390/molecules21040473 27096861 PMC6274295

[B179] Wang M. Carver J. J. Phelan V. V. Sanchez L. M. Garg N. Peng Y. (2016). Sharing and community curation of mass spectrometry data with global natural products social molecular networking. *Nat. Biotechnol.* 34 828–837. 10.1038/nbt.3597 27504778 PMC5321674

[B180] Wang X.E. You J. L. King J. B. Powell D. R. Cichewicz R. H. (2012). Waikialoid A suppresses hyphal morphogenesis and inhibits biofilm development in pathogenic *Candida albicans*. *J. Nat. Prod.* 75 707–715. 10.1021/np2009994 22400916 PMC3338887

[B181] Wang W. J. Li D. Y. Li Y. C. Hua H. M. Ma E. L. Li Z. L. (2014). Caryophyllene sesquiterpenes from the marine-derived fungus *Ascotricha* sp ZJ-M-5 by the one strain-many compounds strategy. *J. Nat. Prod.* 77 1367–1371. 10.1021/np500110z 24878335

[B182] Wang X. R. Sena J. G. Hoover A. R. King J. B. Ellis T. K. Powell D. R. (2010). Chemical epigenetics alters the secondary metabolite composition of guttate excreted by an atlantic-forest-soil-derived *Penicillium citreonigrum*. *J. Nat. Prod.* 73 942–948. 10.1021/np100142h 20450206 PMC2878378

[B183] Wasil Z. Pahirulzaman K. A. K. Butts C. Simpson T. J. Lazarus C. M. Cox R. J. (2013). One pathway, many compounds: heterologous expression of a fungal biosynthetic pathway reveals its intrinsic potential for diversity. *Chem. Sci.* 4 3845–3856. 10.1039/c3sc51785c

[B184] Wijeratne E. M. K. Carbonezi C. A. Takahashi J. A. Seliga C. J. Turbyville T. J. Pierson E. E. (2004). Isolation, optimization of production and structure-activity relationship studies of monocillin I, the cytotoxic constituent of *Paraphaeosphaeria quadriseptata*. *J. Antibiot.* 57 541–546. 10.7164/antibiotics.57.541 15515894

[B185] Wijesekera K. Mahidol C. Ruchirawat S. Kittakoop P. (2017). Metabolite diversification by cultivation of the endophytic fungus *Dothideomycete* sp. in halogen containing media: cultivation of terrestrial fungus in seawater. *Bioorg. Med. Chem.* 25 2868–2877. 10.1016/j.bmc.2017.03.040 28366267

[B186] Williams R. B. Henrikson J. C. Hoover A. R. Lee A. E. Cichewicz R. H. (2008). Epigenetic remodeling of the fungal secondary metabolome. *Org. Biomol. Chem.* 6 1895–1897. 10.1039/b804701d 18480899

[B187] Wu C. Zacchetti B. Ram A. F. van Wezel G. P. Claessen D. Hae Choi Y. (2015). Expanding the chemical space for natural products by *Aspergillus*-*streptomyces* co-cultivation and biotransformation. *Sci. Rep.* 5:10868. 10.1038/srep10868 26040782 PMC4455117

[B188] Wu G. Sun X. Yu G. Wang W. Zhu T. Gu Q. (2014). Cladosins A-E, hybrid polyketides from a deep-sea-derived fungus, *Cladosporium sphaerospermum*. *J. Nat. Prod.* 77 270–275. 10.1021/np400833x 24499327

[B189] Xie L. R. Li D. Y. Li Z. L. Hua H. M. Wang P. L. Wu X. (2013a). A new cyclonerol derivative from a marine-derived fungus *Ascotricha* sp. ZJ-M-5. *Nat. Prod. Res.* 27 847–850. 10.1080/14786419.2012.711327 22840229

[B190] Xie L. R. Li D. Y. Wang P. L. Hua H. M. Wu X. Li Z. L. (2013b). A new 3, 4-seco-lanostane triterpenoid from a marine-derived fungus *Ascotricha* sp. ZJ-M-5. *Acta Pharm. Sin.* 48 89–93. 23600147

[B191] Yang X. L. Awakawa T. Wakimoto T. Abe I. (2013). Induced production of novel prenyldepside and coumarins in endophytic fungi *Pestalotiopsis acaciae*. *Tetrahedron Lett.* 54 5814–5817. 10.1016/j.tetlet.2013.08.054

[B192] Yamazaki H. Rotinsulu H. Narita R. Takahashi R. Namikoshi M. (2015a). Induced production of halogenated epidithiodiketopiperazines by a marine-derived *Trichoderma* cf. *brevicompactum* with sodium halides. *J. Nat. Prod.* 78 2319–2321. 10.1021/acs.jnatprod.5b00669 26439145

[B193] Yamazaki H. Takahashi O. Murakami K. Namikoshi M. (2015b). Induced production of a new unprecedented epitrithiodiketopiperazine, chlorotrithiobrevamide, by a culture of the marine-derived *Trichoderma* cf. *brevicompactum* with dimethyl sulfoxide. *Tetrahedron Lett.* 56 6262–6265. 10.1016/j.tetlet.2015.09.113

[B194] Yang D. Liu F. Yang X. (2017). DNA methyltransferase inhibitor dramatically alters the secondary metabolism of *Pestalotiopsis microspora*. *J. Chin. Pharm. Sci.* 5 355–359. 10.5246/jcps.2017.05.037

[B195] Yang X. L. Huang L. Ruan X. L. (2014). Epigenetic modifiers alter the secondary metabolite composition of a plant endophytic fungus, *Pestalotiopsis crassiuscula* obtained from the leaves of *Fragaria chiloensis*. *J. Asian Nat. Prod. Res.* 16 412–417. 10.1080/10286020.2014.881356 24498889

[B196] Yang X. L. Li Z. Z. (2013). New spiral gamma-lactone enantiomers from the plant endophytic fungus *Pestalotiopsis foedan*. *Molecules* 18 2236–2242. 10.3390/molecules18022236 23434873 PMC6269859

[B197] Yang Y. Fu X. Li L. Zeng Y. Li C. He Y. (2012). Naphthomycins L–N, ansamycin antibiotics from *Streptomyce*s sp. CS. *J. Nat. Prod.* 75 1409–1413. 10.1021/np300109s 22742732

[B198] Yu G. H. Wu G. W. Zhu T. J. Gu Q. Q. Li D. H. (2015). Cladosins F and G, two new hybrid polyketides from the deep-sea-derived *Cladosporium sphaerospermum* 2005-01-E3. *J. Asian Nat. Prod. Res.* 17 120–124. 10.1080/10286020.2014.940330 25081023

[B199] Yuan C. Guo Y. H. Wang H. Y. Ma X. J. Jiang T. Zhao J. L. (2016). Allelopathic polyketides from an endolichenic fungus *Myxotrichum* sp. by using OSMAC strategy. *Sci. Rep.* 6:19350. 10.1038/srep19350 26839041 PMC4738244

[B200] Yuan C. Wang H. Y. Wu C. S. Jiao Y. Li M. Wang Y. Y. (2013). Austdiol, fulvic acid and citromycetin derivatives from an endolichenic fungus, *Myxotrichum* sp. *Phytochem. Lett.* 6 662–666. 10.1016/j.phytol.2013.08.011

[B201] Zhang H. Ruan C. Bai X. Chen J. Wang H. (2018a). Heterocyclic alkaloids as antimicrobial agents of *Aspergillus fumigatus* D endophytic on *Edgeworthia chrysantha*. *Chem. Nat. Compd.* 54 411–414. 10.1007/s10600-018-2365-4

[B202] Zhang H. Zhao Z. Chen J. Bai X. Wang H. (2018b). Tricycloalternarene analogs from a symbiotic fungus *Aspergillus* sp. D and their antimicrobial and cytotoxic effects. *Molecules* 23 855–861. 10.3390/molecules23040855 29642523 PMC6017176

[B203] Zhang L. Niaz S. I. Khan D. Wang Z. Zhu Y. Zhou H. (2017a). Induction of diverse bioactive secondary metabolites from the mangrove endophytic fungus *Trichoderma* sp. (strain 307) by co-cultivation with *Acinetobacter johnsonii* (strain B2). *Mar. Drugs* 15:35. 10.3390/md15020035 28208607 PMC5334615

[B204] Zhang Z. He X. Zhang G. Che Q. Zhu T. Gu Q. (2017b). Inducing secondary metabolite production by combined culture of *Talaromyces aculeatus* and *Penicillium variabile*. *J. Nat. Prod.* 80 3167–3171. 10.1021/acs.jnatprod.7b00417 29144133

[B205] Zhang Z. Chen L. Zhang X. Liang Y. Anjum K. Chen L. (2017c). Bioactive bafilomycins and a new N-Arylpyrazinone derivative from marine-derived *Streptomyces* sp. HZP-2216E. *Planta Med.* 83 1405–1411. 10.1055/s-0043-111897 28571080

[B206] Zhang X. Chen L. Chai W. Lian X. Y. Zhang Z. (2017d). A unique indolizinium alkaloid streptopertusacin A and bioactive bafilomycins from marine-derived *Streptomyces* sp. HZP-2216E. *Phytochemistry* 144 119–126. 10.1016/j.phytochem.2017.09.010 28923323

[B207] Zhang Q. Wang S. Q. Tang H. Y. Li X. J. Zhang L. Xiao J. (2013). Potential allelopathic indole diketopiperazines produced by the plant endophytic *Aspergillus fumigatus* using the one strain-many compounds method. *J. Agric. Food Chem.* 61 11447–11452. 10.1021/jf403200g 24188331

[B208] Zhao Q. Wang G. Q. Chen G. D. Hu D. Li X. X. Guo L. D. (2015). Nodulisporisteroids C-L, new 4-methyl-progesteroid derivatives from *Nodulisporium* sp. *Steroids* 102 101–109. 10.1016/j.steroids.2015.08.004 26254609

[B209] Zheng Q. C. Chen G. D. Kong M. Z. Li G. Q. Cui J. Y. Li X. X. (2013). Nodulisporisteriods A and B, the first 3,4-seco-4-methyl-progesteroids from *Nodulisporium* sp. *Steroids* 78 896–901. 10.1016/j.steroids.2013.05.007 23685090

[B210] Zheng Y. Zhao B. Lu C. Lin X. Zheng Z. Su W. (2009). Isolation, structure elucidation and apoptosis-inducing activity of new compounds from the edible fungus *Lentinus striguellus*. *Nat. Prod. Commun.* 4 501–506. 19475993

[B211] Zhou L. N. Gao H. Q. Cai S. X. Zhu T. J. Gu Q. Q. Li D. H. (2011). Two new cyclic pentapeptides from the marine-derived fungus *Aspergillus versicolor*. *Helv. Chim. Acta* 94 1065–1070. 10.1002/hlca.201000408

[B212] Zhu F. Chen G. Y. Chen X. Huang M. Z. Wan X. Q. (2011). Aspergicin, a new antibacterial alkaloid produced by mixed fermentation of two marine-derived mangrove epiphytic fungi. *Chem. Nat. Compd.* 47 767–769. 10.1007/s10600-011-0053-8

[B213] Zhu F. Lin Y. (2006). Marinamide, a novel alkaloid and its methyl ester produced by the application of mixed fermentation technique to two mangrove endophytic fungi from the south china sea. *Sci. Bull.* 51 1426–1430. 10.1007/s11434-006-1426-4

[B214] Zuck K. M. Shipley S. Newman D. J. (2011). Induced production of *N*-formyl alkaloids from *Aspergillus fumigatus* by co-culture with *Streptomyces peucetius*. *J. Nat. Prod.* 74 1653–1657. 10.1021/np200255f 21667925

